# A surrogate endpoint-based provisional approval causal roadmap, illustrated by vaccine development

**DOI:** 10.1093/biostatistics/kxaf018

**Published:** 2025-06-22

**Authors:** Peter B Gilbert, James Peng, Larry Han, Theis Lange, Yun Lu, Lei Nie, Mei-Chiung Shih, Salina P Waddy, Ken Wiley, Margot Yann, Zafar Zafari, Debashis Ghosh, Dean Follmann, Michal Juraska, Iván Díaz

**Affiliations:** Vaccine and Infectious Disease and Public Health Sciences Divisions, Fred Hutchinson Cancer Center, 1100 Fairview AVE N PO Box 19024, Seattle, WA 98109, United States; Department of Biostatistics, University of Washington, 3980 15th Avenue NE, Box 351617, Seattle, WA 98195, United States; Department of Biostatistics, University of Washington, 3980 15th Avenue NE, Box 351617, Seattle, WA 98195, United States; Department of Public Health and Health Sciences, Bouvé College of Health Sciences, Northeastern University, 30 Leon St, MA 02115, United States; Department of Public Health and Health, University of Copenhagen, Copenhagen K-1353, Denmark; Center for Biologics Evaluation and Research, Food and Drug Administration, 10903 New Hampshire Ave, Silver Spring, MD 20993, United States; Center for Drug Evaluation and Research, Food and Drug Administration, 10001 New Hampshire Avenue, Silver Spring, MD 20993, United States; VA Palo Alto Health Care System, Food and Drug Administration, 3801 Miranda Ave, Palo Alto, CA 94025, United States; Division of Clinical Innovation, National Center for Advancing Translational Sciences, National Institutes of Health, 31 Center Drive MSC 2128 Bethesda, MD 20892, United States; Division of Clinical Innovation, National Center for Advancing Translational Sciences, National Institutes of Health, 31 Center Drive MSC 2128 Bethesda, MD 20892, United States; School of Public Health, UC Berkeley, Forum for Collaborative Research, UCDC Campus, 1608 Rhode Island Avenue NW, Washington, DC 20036, United States; School of Pharmacy, University of Maryland, 20 North Pine Street, Baltimore, MD 21201, United States; Department of Biostatistics & Informatics, Colorado School of Public Health, 13001 E. 17th Place, Aurora, CO 80045, United States; Biostatistics Research Branch, National Institute of Allergy and Infectious Diseases, 5601 Fishers Lane, Rockville, MD 20852, United States; Vaccine and Infectious Disease Division, Fred Hutchinson Cancer Center, 1100 Fairview AVE N, PO Box 19024, Seattle, WA 98109, United States; Department of Biostatistics, New York University Grossman School of Medicine, 180 Madison Ave New York, NY, MD 10016, United States

**Keywords:** causal roadmap, data fusion, group B *Streptococcus*, sensitivity analysis, surrogate endpoint, transportability

## Abstract

For many rare diseases with no approved preventive interventions, promising interventions exist. However, it has proven difficult to conduct a pivotal phase 3 trial that could provide direct evidence demonstrating a beneficial effect of the intervention on the target disease outcome. When a promising putative surrogate endpoint(s) for the target outcome is available, surrogate-based provisional approval of an intervention may be pursued. Following the general Causal Roadmap rubric, we describe a surrogate endpoint-based provisional approval causal roadmap. Based on an observational study data set and a phase 3 randomized trial data set, this roadmap defines an approach to analyze the combined data set to draw a conservative inference about the treatment effect (TE) on the target outcome in the phase 3 study population. The observational study enrolls untreated individuals and collects baseline covariates, surrogate endpoints, and the target outcome, and is used to estimate the surrogate index—the regression of the target outcome on the surrogate endpoints and baseline covariates. The phase 3 trial randomizes participants to treated vs. untreated and collects the same data but is much smaller and hence very underpowered to directly assess TE, such that inference on TE is based on the surrogate index. This inference is made conservative by specifying 2 bias functions: one that expresses an imperfection of the surrogate index as a surrogate endpoint in the phase 3 study, and the other that expresses imperfect transport of the surrogate index in the untreated from the observational to the phase 3 study. Plug-in and nonparametric efficient one-step estimators of TE, with inferential procedures, are developed. The finite-sample performance of the estimators is evaluated in simulation studies. The causal roadmap is motivated by and illustrated with contemporary Group B Streptococcus vaccine development.

## 1. INTRODUCTION

For more than 10,000 rare diseases, no effective treatments are licensed/approved (Fermaglich and Miller 2023). The traditional pathway for approving treatments generates evidence of treatment effectiveness based on one or preferably 2 randomized, controlled phase 3 trials that directly demonstrate benefit on a target outcome that reflects how an individual “feels, functions, or survives” (Fleming and DeMets 1996; Temple 1999; Fleming and Powers 2012). When the target outcome is defined by onset of a rare disease, the phase 3 trial would need a very large sample size (ie hundreds of thousands) to be powered to demonstrate benefit. It is frequently very challenging to garner enough resources to conduct such phase 3 trials.

Yet, promising candidate surrogate endpoints are sometimes available that open an alternative approval pathway to pursue: provisional approval based on phase 3 trials that use the surrogate endpoint as the primary endpoint. By “surrogate endpoint,” we use the definition from a 2016 NIH/FDA workshop (FDA et al. 2016) that was voted by the SPIRIT-SURROGATE/CONSORT-SURROGATE project team as a preferred definition (Ciani et al. 2023): paired to a target outcome of interest, a surrogate endpoint is an intermediate outcome that itself does not reflect “feels, functions, or survives” but can be used as a substitute for the target outcome to reliably provide estimation and inference for the treatment effect on this target outcome. The US FDA’s accelerated approval regulation codifies this pathway (FDA 1992, 2021), which enables provisional approval of a treatment for a serious or life-threatening disease with unmet need based on a sufficiently well validated surrogate endpoint for a target outcome.

Our motivating example is development of vaccines against Group B Streptococcus (GBS), which causes invasive GBS disease (IGbsD) in infants and is a leading cause of young infant death (Vekemans et al. 2019b). No vaccine has been approved to prevent young infant IGbsD. The World Health Organization (WHO) has identified development of a GBS vaccine for immunization during pregnancy as a priority (Kobayashi et al. 2016). While multiple companies are developing maternal GBS vaccines (Vekemans et al. 2019a), no phase 3 vaccine efficacy trial has been conducted, partly because the trial would need to be very large given the low incidence of IGbsD of about 1–3 per 1000 live births (Vekemans et al. 2019b). Therefore, the GBS vaccine field is currently pursuing a provisional approval pathway based on antibody markers measured in cord blood that have been shown to strongly inversely correlate with IGbsD in natural history studies (Madhi et al. 2021, 2023; Dangor et al. 2023). The US FDA’s Vaccine Advisory Board in May of 2018 concluded that one of these antibody markers, the concentration of IgG antibody binding to GBS proteins, is reasonably likely to predict vaccine efficacy of capsular polysaccharide GBS vaccines against IGbsD, and recommended a provisional approval pathway based on this surrogate endpoint (Gilbert et al. 2022). The EMA Guideline on clinical evaluation of vaccines EMEA/CHMP/VWP/164653/05 Rev. 1 also stated support for this approval pathway when target outcome efficacy trials are not feasible.

For decades, regulators have authorized or approved new vaccines based on a phase 3 trial with an immunological biomarker surrogate as the primary endpoint, for example, to update influenza and COVID-19 vaccines to contain new viral strains. However, to the best of our knowledge, in all such instances traditional disease-outcome phase 3 trials preceded the product approvals, highlighting the innovation in pursuing a new approach to provisionally approving products for prevention of (very) rare diseases.

The Causal Roadmap is a general 7-step framework for pursuing answers to causal questions (Petersen and van der Laan 2014; Dang et al. 2023). In this article, we apply this framework to define a Causal Roadmap for the provisional approval objective contextualized above, using contemporary GBS vaccine development as a running example. Our scope considers contexts meeting all 3 of the following conditions: (i) no randomized, controlled phase 3 trials have been conducted of the candidate treatment of interest and it has proven formidable to conduct such phase 3 trials such that none are expected on the horizon, (ii) one or more prospective observational studies have been conducted in untreated persons that assess the relationship of one or more candidate surrogate endpoints with a target outcome of interest, and (iii) a treatment for the disease is being developed with a provisional approval strategy via a pivotal randomized, controlled phase 3 trial with the candidate surrogate endpoint as the primary endpoint.

In addition to this phase 3 trial being an actual trial that is well-powered to study the treatment effect on the surrogate endpoint, we envisage a hypothetical target trial (Hernán and Robins 2016; Hernán et al. 2022)—which would enroll a vastly larger sample size and hence power the trial to assess the treatment effect on the target outcome. Based on a harmonized data set from an observational study (or studies) to learn a surrogate and from a phase 3 trial to apply the surrogate, a statistical approach is needed to estimate the treatment effect on the target outcome in the target trial with an estimated uncertainty interval (EUI) codified in some fashion. We consider that the primary success criterion for provisional approval may be defined by this EUI lying above a pre-specified minimum lower bound of the treatment effect on the target outcome determined through iterative deliberations with regulators, and may also require a minimal point estimate.

As such, our objective is to apply the Causal Roadmap to develop a process, culminating in a statistical analysis plan encompassing the observational and phase 3 studies, for transparently obtaining the EUI and point estimates that determine success vs. failure of meeting provisional approval criteria.

Current GBS vaccine development will serve as our illustrative example in the article. Indeed, multiple vaccine developers are pursuing this approach that characterizes an antibody surrogate endpoint in multiple sero-epidemiological natural history observational studies and then conducts a phase 3 surrogate endpoint trial to evaluate qualification against provisional approval success criterion. Envisaged as a target trial, the primary objective of the phase 3 trial is estimation/inference of vaccine efficacy against IGbsD for the phase 3 study population based on the surrogate defined from data analysis of the observational studies. Our Surrogate Based Provisional Approval Causal Roadmap provides one way to statistically answer this primary objective. As of February 2025, a vaccine developer is designing a phase 3 trial using the provisional approval pathway, with the Causal Roadmap supporting a statistical analysis plan for the approval objective.

The problem we address is related to the transportability literature, which has focused on extending causal inferences from one or more randomized trials to a target population represented by an observational study sample (eg Cole and Stuart 2010; Westreich et al. 2017; Buchanan et al. 2018; Li and Luedtke 2023). For example, Rudolph and Laan (2017), Dahabreh et al. (2020), and Dahabreh et al. (2023b) developed methods including robust targeted maximum likelihood, g-formula, inverse probability weighting, and combined double-robust methods for extending causal inferences about a point treatment effect from a randomized trial to a target population of non-participants, based on all the data from the randomized trial and a sample of baseline covariates from the target population. In this application, the utility of the baseline covariates is to correct for bias from treatment effect modifiers influencing participation in the randomized trial. Dahabreh et al. (2023b) is especially germane because they developed methods for sensitivity analysis via bias functions that provide conservative lower-bound inferences for the treatment effect in the target population, fitting the essential requirement of our provisional approval objective. However, the present work departs from Dahabreh et al. (2023b) in 2 main ways. First, in our problem context, knowledge learned from an observational study (with no treatment) is applied to make inferences about a treatment effect in a new target population that is studied in a randomized trial (reversing the role of the randomized and observational study). Second, knowledge is extended based on the relationship between an intermediate response surrogate endpoint and baseline covariates on the target outcome, instead of only considering baseline covariates. The implication is that different causal identifiability assumptions are needed to license valid inferences on the causal treatment effect in the target population. Another implication is that the notion of a valid surrogate endpoint is important for our problem, where there is a large literature on surrogate endpoint evaluation based on target outcome randomized trials (eg Alonso et al. 2015). However, because in our scenario no phase 3 target outcome randomized trial has yet been done, evidence for the appropriateness of the surrogate will need to come from other sources, and we describe below the requirements for the surrogate endpoint to buttress the desired correct inferences.

Athey et al. (2024) considered a similar general statistical problem, independently developing methods of inference for the same treatment effect target causal parameter of interest, based on the same collected data. However, Athey et al. assume observational study participants have treatment missing or unknown, whereas we assume they all have the control condition treatment. Under both a perfect comparability assumption and a perfect surrogacy assumption (special cases of A4 and A6 below), the identifiability results and nonparametric efficient influence function are equivalent in their work and the present manuscript. However, with less than perfect comparability or surrogacy, the results differ.

In particular, the present article focuses on conservative inference (under imperfect comparability and surrogacy) as this is a requirement of the provisional approval application. Another difference is that the present article considers some practical challenges arising in the application, including missing data on the surrogate(s) and right-censoring of the target outcome. Section SA compares the methods of the 2 manuscripts.

## 2. NOTATION AND DATA SOURCES: OBSERVATIONAL STUDY AND PHASE 3 STUDY

Our notation is similar to Dahabreh et al. (2023b), except using $ Z $ instead of $ S $ to denote study. We consider a single observational study. Let $ Z\,=\,1 $ ($ Z\,=\,0 $) indicate enrollment into the observational (phase 3) study. The observational (phase 3) study enroll $ n_{obs} $ ($ n_{RCT} $) individuals, with covariates $ X $ measured at enrollment and intermediate outcomes $ S $ measured after enrollment and by the fixed visit $ \tau $ post-enrollment (in many applications $ S $ is biomarkers measured from a blood sample drawn at time $ \tau $). In study $ Z\,=\,1 $ participants are followed after $ \tau $ over a fixed period through time $ t_{0} $ for whether the target outcome occurs: $ Y\,=\,I(T\leq t_{0}) $ where $ T $ is the time from $ \tau $ to the target outcome failure event and $ I(B) $ is the indicator of an arbitrary event $ B $; also let $ C $ be the time from $ \tau $ until right-censoring, with $ \tilde{T}=min(T, C) $ and $ \Delta $ the indicator of observed failure after $ \tau $ by $ t_{0} $, $ \Delta=I(T\leq C) $. Follow-up through $ t_{0} $ without the event means $ \tilde{T}=C $ and $ \Delta\,=\,0 $. In many applications, $ S $ is only meaningfully defined if the participant did not experience the target outcome by $ \tau $, in which case such participants are excluded for the purpose of surrogate endpoint evaluation. The issue of intercurrent events is addressed at the end of the section on Step 5.

For study design, $ \tau $ is selected for viability of defining a surrogate endpoint based on measurements of $ S $ up to and including time $ \tau $, where broad inter-individual variability in $ S $ across participants in the $ Z\,=\,0 $ and $ Z\,=\,1 $ studies improves precision for estimation of the treatment effect parameter. It is desirable to select $ \tau $ close to enrollment, to attain the practical advantage of a surrogate endpoint to facilitate shorter studies.

Given that the target outcome $ Y\,=\,1 $ is rare, for resource efficiency $ S $ in the $ Z\,=\,1 $ observational study (and perhaps also the $ Z\,=\,0 $ phase 3 trial) is measured in a random sample of participants, such as through 2-phase case-cohort sampling. Let $ \epsilon_{S} $ be the indicator that $ S $ is measured. Let $ A $ be the indicator that a participant receives treatment; all participants in the observational study have $ A\,=\,0 $ and phase 3 participants are randomized to $ A\,=\,1 $ or the control condition $ A\,=\,0 $ such as placebo.

To simplify exposition, we assume all enrolled participants in both studies attend the visit $ \tau $ at which $ S $ is measured without experiencing the target outcome. Section SB considers relaxation of this assumption. In total, the composite data set consists of $ n\,=\,n_{obs}+n_{RCT} $ observations, with $ n_{obs} $ iid observations $ (X_{i},Z_{i}=1, A_{i}=0 , \epsilon_{Si},\epsilon_{Si}S_{i},\tilde{T}_{i},\Delta_{i}) $ and $ n_{RCT} $ iid observations $ (X_{i},Z_{i}=0, A_{i},\epsilon_{Si},\epsilon_{Si}S_{i}) $, where $ Y_{i}=I(T_{i}\leq t_{0}) $ has a known value if $ \tilde{T}_{i}=t_{0} $ and $ \Delta_{i}=0 $ or if $ \tilde{T}_{i}\leq t_{0} $ and $ \Delta_{i}=1 $, and the notation $ \epsilon_{Si}S_{i} $ means that $ S_{i} $ is observed if $ \epsilon_{Si}=1 $. In typical applications $ \tilde{T}_{i} $ and $ \Delta_{i} $ are also collected in the phase 3 study; yet an essential feature of our problem set-up is very few observed $ Y_{i}=1 $ events are expected in the phase 3 study (<10 in phase 3 GBS vaccine studies), and all our results (identifiability and estimation) do not make use of any $ \tilde{T}_{i} $ and $ \Delta_{i} $ values of phase 3 participants.

The relevant $ S $ are intermediate outcomes that, based on domain knowledge, can potentially predict $ Y $ and may be connected to treatment-efficacy mechanisms and hence possibly contribute to a surrogate endpoint. The surrogate index $ g(x, s):=P(Y\,=\,1|X\,=\,x, Z\,=\,1, A\,=\,0, S\,=\,s) $ is the central ingredient for estimating treatment efficacy in the phase 3 study, where the observational study is used to develop an optimal estimator for $ g(X, S) $. Price et al. (2018) proposed that an optimal estimator of $ g(X, S) $—a so-called estimated optimal surrogate (EOS)—be considered for use as a surrogate endpoint. The EOS has a biomedically-relevant interpretation by being on the scale of the absolute risk of the target outcome. The analysis of the observational study for estimation of $ g(X, S) $ may consider many different input variable sets $ (X, S) $ and different ways of entering the variables into models, seeking empirical learning of a most promising EOS. A selected EOS from the observational study is used in the estimators of treatment efficacy in the phase 3 study.

In the phase 3 study, it is not required to measure all the components of $ X $ and $ S $ that were measured in the observational study; it is only required to measure the components that are used in the estimator of $ g(X, S) $ in the observational study. Therefore, empirical learning of $ g(X, S) $ in the observational study can be used to winnow down to a subset of $ (X, S) $ variables to measure in the phase 3 study to potentially save resources and focus on a more parsimonious surrogate. Justification for this would include learning that not all $ (X, S) $ are needed for obtaining an EOS, and evidence that the selected $ (X, S) $ are sufficient for meeting the causal assumptions (listed in Step 4) and not widening estimated uncertainty intervals.

## 3. A SURROGATE ENDPOINT-BASED PROVISIONAL APPROVAL CAUSAL ROADMAP

In this section, we apply each step of the Causal Roadmap to constitute a version of the roadmap for the surrogate endpoint-based provisional approval application with its objective inference on TE in the phase 3 study population using both the observational and phase 3 data.

### 3.1. Step 1: Specify the causal model based on available knowledge of the context and studies

**Fig. 1.**
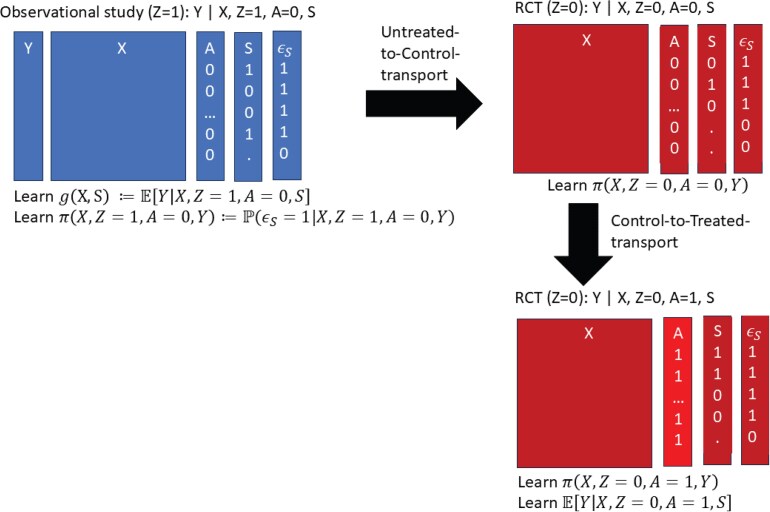
Transportability: Learning about risk of a target outcome $ Y $ for treatment $ A\,=\,1 $ in a phase 3 target trial $ Z\,=\,0 $ (that is very under-powered to directly assess the treatment effect on $ Y $) using information on $ (X, S)- $conditional risk of untreated $ A\,=\,0 $ participants in an observational study $ Z\,=\,1 $ (surrogate index), the probability of sampling $ S $, $ \pi(X, Z\,=\,1, A, Y) $, $ (X, A, S) $ in the phase 3 trial $ Z\,=\,0 $, and Comparability and Surrogacy assumptions A4 and A6. A bias function is used in each step of the transport to achieve lower-bound estimation of treatment efficacy in the phase 3 trial.

In this step, the researcher specifies available knowledge about the candidate surrogate $ S $ as a valid surrogate for the target outcome of interest as pertinent to the specific treatment under development vs. the control arm. This knowledge will be needed for devising an approach to transporting knowledge learned about the relationship of $ (X, Z\,=\,1, A\,=\,0, S) $ with $ Y $ in the observational study (in a causal sense considered in 2 parts) to 2 new settings lacking direct empirical data. Figure 1 diagrams the 2 parts. Part 1, “Untreated-to-Control-transport across studies,” transports the relationship of $ (X, S) $ with $ Y $ from the (all) untreated observational study population ($ Z\,=\,1, A\,=\,0 $) to the untreated/placebo arm of the phase 3 target trial ($ Z\,=\,0, A\,=\,0 $). Part 2, “Control-to-Treated-transport in the phase 3 trial,” transports the relationship of $ (X, S) $ with $ Y $ from the untreated/placebo arm of the phase 3 target trial ($ Z\,=\,0, A\,=\,0 $) to the treated arm of the phase 3 target trial ($ Z\,=\,0, A\,=\,1 $).

Untreated-to-Control-transport addresses the need to bridge knowledge of outcomes learned for the untreated in the observational study to the untreated in the phase 3 study, accounting for potentially different distributions of baseline variables and of the candidate surrogate. Control-to-Treated-transport addresses the need to take the bridged knowledge of outcomes for the untreated in the phase 3 study to the treated in the phase 3 study, addressing the issue that the candidate surrogate may relate to the target outcome differently in the treated and the untreated. Conceptualizing the bridging in 2 distinct parts has advantage of aiding examination of assumptions and designing interpretable sensitivity analyses.

**Fig. 2.**
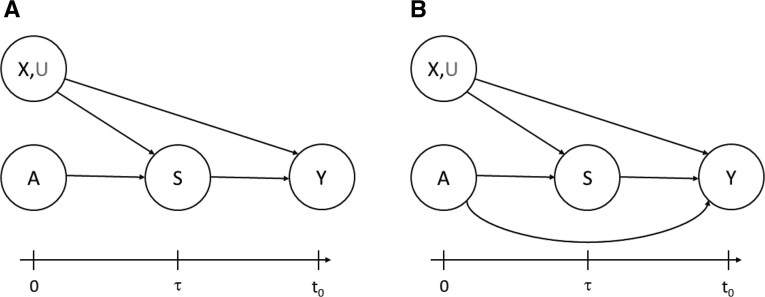
Causal models for the relationship of treatment $ A $, baseline covariates $ X $, surrogate $ S $, and target outcome $ Y $ in the phase 3 target trial $ Z\,=\,0 $: (A) Model expressing a perfect surrogate ($ u^{CT}(X, S)=0 $), (B) Model expressing an imperfect surrogate via a non-zero bias function $ u^{CT}(X, S) $ where $ A $ affects $ Y $ through mechanisms not captured by $ S $. $ U $ indicates potential unmeasured confounders of the effect of $ S $ on $ Y $.

Typical pre-requisite knowledge for a candidate surrogate endpoint to hold promise for being able to accomplish the objective include (i) the endpoint is measured accurately and precisely, with low measurement error; (ii) the endpoint has broad inter-individual variability across treated and untreated persons (for the populations studied); (iii) the endpoint is strongly associated with the target outcome in natural history contexts including the observational study $ Z\,=\,1 $ within level of $ X $; and (iv) the endpoint is connected to putative causal pathway mechanisms of effectively preventing (or treating) the disease. Evidence for (iv) can be most compelling when generated from studies that directly manipulate/assign the surrogate endpoint such as experiments that can be conducted in animal models. Figure 2 shows 2 causal models for the surrogate endpoint in the randomized $ Z\,=\,0 $ study. Panel (A) expresses a perfect surrogate causal model, defined by equal conditional means for treatment assignment $ A\,=\,1 $ vs. $ A\,=\,0 $, expressed as assumption A6 below with bias function $ u^{CT}(X, S)=0 $. Panel (B) expresses an imperfect surrogate causal model, where the treatment $ A $ has an additional effect on $ Y $ not mediated through $ S $, a situation where A6 is needed with non-zero bias function to account for the incomplete mediation.

Measuring the same $ X $ and $ S $ at the same time points in the same way in the $ Z\,=\,1 $ and $ Z\,=\,0 $ studies is basic knowledge informing Untreated-to-Control-transport, as is designing the phase 3 study to take place in a similar population as the observational study. For informing Control-to-Treated-transport, if phase 3 target-outcome trials were available, then a large literature of statistical methods for surrogate endpoint evaluation could be applied (eg Buyse et al. 2016; Xie et al. 2019; Weir and Taylor 2022), where, for example, assessing the nearness of controlled direct effects (at $ S\,=\,s $) of $ A $ on $ Y $ (which compare risk under assignment to $ A\,=\,1 $ vs. $ A\,=\,0 $ holding $ S\,=\,s $ fixed for both $ A\,=\,1 $ and $ A\,=\,0 $) to zero would be valuable knowledge for specifying the causal model (Joffe and Greene 2009; Gilbert et al. 2023). The surrogate evaluation literature includes individual- and trial-level evaluation (Buyse et al. 2016), where our approach relies on individual-level surrogacy given that in our set-up there does not exist a series of studies with treatment effects assessable for both the surrogate and target outcomes. However, trial-level surrogacy evaluation information may be available from pre-clinical animal model studies, which could improve the evidence package for use of a surrogate for provisional approval. In any case, to justify the surrogate researchers will need to leverage domain knowledge of the specific treatment $ A $ and specific outcomes $ S $ and $ Y $.

### 3.2. Step 2: Define the causal parameter of interest

With $ S(a) $ and $ Y(a) $ potential outcomes of the surrogate and target endpoints under randomization assignment $ a\,=\,0,1 $ in the phase 3 trial $ Z\,=\,0 $, the causal parameter of interest is a contrast of $ E[Y(1)|Z\,=\,0] $ and $ E[Y(0)|Z\,=\,0] $, such as the average treatment effect $ E[Y(1)|Z\,=\,0]-E[Y(0)|Z\,=\,0] $. To fit the GBS vaccine case study, throughout we consider the multiplicative vaccine efficacy or treatment efficacy (TE) contrast TE = $ 1-E[Y(1)|Z\,=\,0]/E[Y(0)|Z\,=\,0]. $ The development considers estimation and inference for each of $ E[Y(1)|Z\,=\,0] $ and $ E[Y(0)|Z\,=\,0] $, implying it applies for any preferred contrast of $ E[Y(1)|Z\,=\,0] $ and $ E[Y(0)|Z\,=\,0] $. For (very) rare diseases, inferences about TE are more precise when TE is an additive contrast compared to a multiplicative contrast, suggesting merit in exploring whether regulators would accept an additive contrast. However, typically multiplicative contrasts are used and required because they are less influenced by the background event rate and are considered to be more useful for transporting knowledge of efficacy across settings.

We focus on the phase 3 trial population as the target population for inference because, for our provisional approval objective, the normative approach in practice defines the success criterion (indicating qualification for provisional approval) in terms of a causal treatment effect on the surrogate endpoint in the phase 3 trial. In vaccine studies, this approach, frequently used by the US FDA, is referred to as immunobridging (eg Krause et al. 2022). The randomization in the phase 3 trial not only facilitates achieving valid estimation/inference for an average treatment effect causal parameter but also is a key factor for attaining widespread credibility of the results that is important for uptake of the provisionally approved treatment. Alternatively, the observational study population could be the target of inference (studying a contrast of $ E[Y(1)|Z\,=\,1] $ vs. $ E[Y(0)|Z\,=\,1] $), which will frequently be of interest in its own right. The methods in this manuscript can be readily adapted to make inference for the observational study population.

### 3.3. Steps 3 and 4: Translate the causal parameter into a statistical estimand; identify conditions under which the statistical estimands equal their corresponding causal parameters

After listing identifiability conditions (Step 4), we describe how under these assumptions the statistical estimands equal their corresponding causal parameters (Step 3). The conditions use 2 known user-specified bias functions for sensitivity analysis: $ u^{UC}(x, s):=E[Y(0)|X\,=\,x, Z\,=\,1, S(0)=s]-E[Y(0)|X\,=\,x, Z\,=\,0, S(0)=s] $ and $ u^{CT}(x, s):=E[Y(1)|X\,=\,x, Z\,=\,0, S(1)=s]-E[Y(0)|X\,=\,x, Z\,=\,0, S(0)=s] $, with UC standing for Untreated-to-Control-transport and CT standing for Control-to-Treated-transport. The bias functions can be written as statistical estimands:


1 \begin{align*} u^{UC}(x, s) = E[Y|X=x, Z=1, A=0, S=s]-E[Y|X=x, Z=0, A=0, S=s],\end{align*}

2 \begin{align*} u^{CT}(x, s) = E[Y|X=x, Z=0, A=1, S=s]-E[Y|X=x, Z=0, A=0, S=s],\end{align*}

where (i) holds by causal consistency A1 and because $ A\,=\,0 $ for all $ Z\,=\,1 $ participants, and (ii) holds by A1 and because the $ Z\,=\,0 $ study is randomized (A2).

If the phase 3 study collects data on $ Y $, then both bias functions are identified from the observed data. However, even with such data collection the study would have very low precision to estimate $ u^{UC}(x, s) $ and $ u^{CT}(x, s) $. Therefore, the bias functions are treated as fixed user-specified sensitivity parameters, the former expressing a degree of residual differences in background risk after accounting for differences in $ (X, S) $ and the latter expressing the quality of the candidate surrogate $ S $. The special case $ u^{UC}(x, s)=0 $, which specifies no residual bias, and the special case $ u^{CT}(x, s)=0 $, which specifies an ideal/perfect surrogate, are not of central interest for our provisional approval application where lower-bound estimation of TE is a central requirement.

We list the identifiability conditions (Step 4), describe how they link the causal parameters to statistical estimands (Step 3), and then discuss the identifiability conditions.

### Step 4: Identifiability assumptions

A1 *Consistency:* For each individual $ i $ in the phase 3 study $ Z=0 $, the observable surrogate and observable target outcome under treatment $ A_{i}=a $ equals that individual’s counterfactual surrogate and counterfactual target outcome under the same treatment, that is $ A_{i}=a $ implies $ S_{i}=S_{i}(a) $ and $ Y_{i}=Y_{i}(a) $ for $ a=0,1 $. For each individual $ i $ in the observational study $ Z=1 $, the same results attain for $ a=0 $.
A2 *Conditional mean exchangeability in the phase 3 trial (over $ A $):* Among randomized individuals (in study $ Z=0 $), the potential target outcome mean under treatment $ a $ is independent of treatment, conditional on baseline covariates $ X $, that is $ E[Y(a)|X=x, Z=0, A=a]=E[Y(a)|X=x, Z=0] $ for each $ a=0,1 $ and every $ x $ with positive density $ f_{(X, Z)}(x, z=0) > 0 $.
A3 *Positivity of treatment assignment in the phase 3 study:* In the phase 3 study, the probability of being assigned each treatment $ a=0,1 $, conditional on the covariates $ (X, S) $ used in assumption A2, is bounded away from 0 and 1, that is there exist constants $ c_{0},c_{1}\in(0,1) $ such that $ c_{0} < P(A=a|X=x, Z=0, S=s) < c_{1} $ for each $ a=0,1 $ and every $ (x, s) $ with positive joint density $ f_{(X, Z, S)}(x, z=0, s) > 0 $.
A4 **Comparability Assumption—** *Conditional mean exchangeability of the untreated with bias function from the observational study to the phase 3 study (exchangeability over $ Z $ in the untreated $ A=0 $):* $ E[Y(0)|X=x, Z=1, S(0)=s]=E[Y(0)|X=x, Z=0, S(0)=s]+u^{UC}(x, s) $ for every $ (x, z=0, a=0, s) $ with positive joint density $ f_{(X, Z, A=0, S)}(x, z=0, a=0, s) > 0 $.
A5 *Positivity of being enrolled in the observational study:* The probability of enrolling in the observational study, conditional on the covariates $ (X, S) $ used in assumptions A2, A3, and A4, is bounded away from 0 and 1, that is there exist constants $ k_{0},k_{1}\in(0,1) $ such that $ k_{0} < P(Z=1|X=x, S=s) < k_{1} $ for every $ (x, s) $ with positive joint density $ f_{(X, S)}(x, s) > 0 $.
A6 **Surrogacy Assumption—** *Correct specification of the CT bias function that provides exchangeability over $ A $ in the phase 3 study:* $ E[Y(1)|X=x, Z=0, S(1)=s]=E[Y(0)|X=x, Z=0, S(0)=s]+u^{CT}(x, s) $ for every $ (x, z=0, a=0, s) $ with positive joint density $ f_{(X, Z=0, A=0, S)}(x, z=0, a=0, s) > 0 $.
A7 *Missing at random surrogate in both studies:* The probability of observing $ S $ does not depend on the value of $ S $, that is with
  3 \begin{align*}\pi(X, Z, A,\tilde{T},\Delta):=P(\epsilon_{S}=1|X, Z, A,\tilde{T},\Delta),\end{align*}

$ \pi(X, Z=1, A=0, S,\tilde{T},\Delta)=\pi(X, Z=1, A=0 , \tilde{T},\Delta) $ and $ \pi(X, Z=0, A, S , \tilde{T},\Delta)=\pi(X, Z=0, A,\tilde{T},\Delta) $. Note that because target outcome data $ (\tilde{T},\Delta) $ are not available/used for the $ Z=0 $ study participants, $ \pi(X, Z=0, A,\tilde{T},\Delta) $ actually equals $ P(\epsilon_{S}=1|X, Z=0, A) $; we leave this tacit in the notation.
A8 *Random right-censoring of the target outcome in the observational study:* In the $ Z=1 $ observational study, no participants are right-censored through the final time point $ t_{0} $, or, if there is, right-censoring is random within levels of baseline covariates, i.e., $ T\perp C|X, Z=1, A=0 $.

### 3.5. Step 3: Connecting the causal parameters to the statistical estimands

Define $ g(X, S):=E[Y|X, Z\,=\,1, A\,=\,0, S] $. For the Untreated-to-Control-transport, the following equation holds under A1, A4, A5, A8 (proof in Section SC):


4 \begin{align*} E[Y(0)|Z=0]=E\big\{E[g(X, S)|X, Z=0, A=0]|Z=0\big\}-E\big\{E[\mu^{UC}(X, S)|X, Z=0, A=0]|Z=0\big\}.\end{align*}

The last step establishing identifiability addresses the fact that $ S $ is only measured in a random sample. From Rose and van der Laan (2011), $ g(X, S) $ is identified as:


5 \begin{align*} g=\arg\min\limits_{\tilde{g}}E\left[\frac{\epsilon_{S}L(\tilde{g})(X, Z= 1, S,\tilde{T},\Delta)\,}{P(\epsilon_{S}=1|X, Z=1, A=0 , \tilde{T},\Delta)}\bigg|\, Z=1, A=0\right],\end{align*}

where $ L(\tilde{g})(\cdot) $ is the loss function as if $ S $ had been measured for all $ Z\,=\,1 $ participants.

For the Control-to-Treated-transport, the following equation holds under A1–A6, A8:


6 \begin{align*} E[Y(1)|Z=0] & = E\big\{E[g(X, S)|X, Z=0, A=1]|Z=0\big\}\nonumber\\&\quad + E\big\{E[\mu^{CT}(X, S)-\mu^{UC}(X, S)|X, Z=0, A=1]|Z=0\big\}.\nonumber\end{align*}

Then Equation (5) completes the identifiability result for $ E[Y(1)|Z\,=\,0] $.

The Equations (4) and (6) are designed such that $ g(X, S) $, which conditions on being in the observational study $ Z\,=\,1 $ and is the only direct source for learning about the surrogate-target outcome relationship, is the key regression for estimating treatment efficacy in the new setting $ Z\,{=}\,0 $. If both bias functions are zero, then high treatment efficacy TE is attained if both (i) $ g(X, S) $ is monotone non-increasing in $ S $ and decreases by a large amount; and (ii) the distribution of the surrogate $ S $ in the $ Z\,=\,0 $ study is stochastically much higher in the treated than untreated control $ A\,=\,1 $ vs. $ A\,=\,0 $. Vice versa, either $ g(X, S) $ independent of $ S $ (ie $ g(X, S)=g(X) $ such that $ S $ is not a correlate of risk in the $ Z\,=\,1 $ study), or $ S|X, Z\,=\,0, A\,=\,1=^{d}S|X, Z\,=\,0, A\,=\,0 $ (no treatment effects on the surrogate in the $ Z\,=\,0 $ study), imply TE = 0. Section SC derives (4) and (6), detailing the assumptions needed for each step.

A1–A5 are similar to identifiability conditions (I)–(V) respectively of Dahabreh et al. (2023b), except in our problem the target setting for transporting the treatment effect ($ Z\,=\,0 $) is the randomized trial, not the reverse as in Dahabreh et al. (2023b), and A3, A4, A5 involve intermediate outcomes $ S $ that Dahabreh et al. (2023b) did not consider. Also, in A4 we include the bias function $ u^{UC}(X, S) $ in the assumption, where setting $ u^{UC}(X, S)=0 $ yields the standard conditional mean exchangeability assumption across studies for the untreated (Rudolph and Laan 2017; Dahabreh et al. 2023b). A5 is an overlap condition making possible borrowing knowledge on background risk in the observational study to apply to the untreated control arm in the phase 3 study.

A6 is new for our problem, which exchanges/transports knowledge of the relationship of the surrogate and target outcome from the untreated control arm to the treated. Setting $ u^{CT}(X, S)\,{=}\,0 $ defines the “complete mediation” condition that is a causal version of Prentice’s (1989) third criterion for a valid surrogate endpoint that was also considered in Price et al. (2018) (Theorem 3). Specifying non-zero $ u^{CT}(X, S) $ provides a sensitivity analysis acknowledging imperfection of the surrogate. Use of A6 relies on A3 which requires that the distribution of the surrogate endpoint has overlapping support across the treated and control arms of the phase 3 trial. A7 and A8 address missing data on $ S $ and missing data on $ Y $, respectively.

For our purpose of informing provisional approval, it is of interest to specify both $ u^{UC}(X, S) $ and $ u^{CT}(X, S) $ to make it more difficult to meet the approval success criterion, for inculcating conservatism to lower the risk of provisionally approving a treatment that later proves to have poor performance against the target outcome.

### 3.6. Step 5: Estimate the statistical estimand

Estimation requires a technique for accommodating missing data for $ S $ in both studies, as well as for right-censoring of $ T $ before $ t_{0} $ in the $ Z\,=\,1 $ study. We address missing values of $ S $ with inverse probability sampling (IPS) weighted complete-case estimation. Because in both studies $ Z\,=\,0,1 $ the investigator designs a plan for sampling the set of participants from whom to measure $ S $, with the sampling design depending on $ (X, Z\,=\,1, A\,=\,0 , \tilde{T},\Delta) $ for the observational study and on $ (X, Z\,=\,0, A) $ for the randomized study, it is generally attainable to correctly model $ \pi(\cdot) $ [defined at (3)]. The statistical estimands [RHSs of (4) and (6)] that link to the causal parameters $ E[Y(0)|Z\,=\,0] $ and $ E[Y(1)|Z\,=\,0] $ under the causal assumptions can be written as


7 \begin{align*}\theta_{a}:=E\big\{E[g^{*}_{a}(X, S)|X, Z=0, A=a]|Z=0\big\},a=0,1 , \end{align*}

where $ g^{*}_{0}(x, s):=E[Y-\mu^{UC}(X, S)|X\,=\,x, Z\,=\,1, A\,=\,0, S\,=\,s] $ recovers the identification formula for $ E[Y(0)|Z\,=\,0] $ and $ g^{*}_{1}(x, s):=E[Y+\mu^{CT}(X, S)-\mu^{UC}(X, S)|X\,=\,x, Z\,=\,1, A\,=\,0, S\,=\,s] $ recovers it for $ E[Y(1)|Z\,=\,0] $.

#### 3.6.1. Plug-in estimator

Define the plug-in estimator for each $ a\,=\,0,1 $ as


8 \begin{align*}\widehat{\theta}_{a , {\tiny plug-in}}=\frac{1}{n_{RCT}}\sum\limits_{i=1}^{n}(1-Z_{i})\widehat{E}[{\widehat{g}^{*}_{a}(X, S)}|X_{i},Z_{i}=0, A_{i}=a],\end{align*}

where $ \widehat{g}^{\,*}_{a}(X, S) $ is obtained using the loss function in Equation (5), which can be implemented with any regression estimator using weights $ 1/\widehat{\pi}(X_{i},Z_{i},A_{i},\tilde{T}_{i},\Delta_{i}) $. If the bias functions $ u^{UC}(X, S) $ and $ u^{CT}(X, S) $ are set to known constants $ u^{UC} $ and $ u^{CT} $, then $ \widehat{g}^{*}_{a}(X, S) $ is obtained as $ \widehat{g}(X, S)-u^{UC} $ and $ \widehat{g}(X, S)+u^{CT}-u^{UC} $ for $ a\,=\,0 $ and $ a\,=\,1 $, respectively, based on a single regression estimator $ \widehat{g}(X, S) $. The outer expectation estimate $ \widehat{E}[\cdot] $ can be obtained for $ a\,=\,0 $ by regressing $ \widehat{g}^{*}_{0}(X, S) $ on $ X_{i} $ including individuals with $ Z_{i}=0, A_{i}=0 $ and $ \epsilon_{S, i}=1 $ and including the IPS weights $ 1/\widehat{\pi}(X_{i},Z_{i},A_{i},\tilde{T}_{i},\Delta_{i}) $. Similarly, for $ a\,=\,1 $, we can estimate the outer expectation $ \widehat{E}[\cdot] $ by performing IPS-weighted regression of $ \widehat{g}^{*}_{1}(X, S) $ on $ X_{i} $ including individuals with $ Z_{i}=0, A_{i}=1 $ and $ \epsilon_{S, i}=1 $.

After the section on the nonparametric efficient one-step estimator, we restrict to constant bias functions, thus only requiring estimation of a single outcome regression $ g(X, S) $, and yielding an easily interpreted sensitivity analysis.

#### 3.6.2. Nonparametric efficient one-step estimator

To develop an efficient estimator, we follow a general approach outlined by Rose and van der Laan (2011). First, assume a hypothetical data structure with $ \epsilon_{S}=1 $ with probability one. We define the efficient influence function (EIF) $ \varphi_{a}(O;\eta) $ for the case that $ Y $ is always observed, equal to


9 \begin{align*}& \frac{I(Z=1)}{P(Z=0)}\frac{I(A=0)}{P(A=a|X, Z=0)}\frac{P(Z=0, A=a|X ,S)}{P(Z=1, A=0|X, S)}\times\{Y+a\mu^{CT}(X, S)-\mu^{UC}(X, S)-g^{*}_{a}(X, S)\}\nonumber\\&\quad +\frac{I(Z=0)}{P(Z=0)}\frac{I(A=a)}{P(A=a|X, Z=0)}\times\{g^{*}_{a}(X, S)-E[g^{*}_{a}(X, S)|X, Z=0, A=a]\}\nonumber\\&\quad +\frac{I(Z=0)}{P(Z=0)}\{E[g^{*}_{a}(X, S)|X, Z=0, A=a]-\theta_{a}\},\end{align*}

where $ \eta $ is used to denote the nuisance parameters appearing in the EIF. Results in Rose and van der Laan (2011) show that, when $ P(\epsilon_{S}=1) < 1 $, the EIF can be constructed as:


10 \begin{align*}\phi_{a}(O;\eta) & = \frac{\epsilon_{S}}{P(\epsilon_{S}=1|X, Z, A,\tilde{T},\Delta)}\varphi(O;\eta)\nonumber\\&\quad +\left\{1-\frac{\epsilon_{S}}{P(\epsilon_{S}=1|X, Z, A,\tilde{T},\Delta)}\right\}E[\varphi(O;\eta)|\epsilon_{S}=1, X, Z, A,\tilde{T},\Delta].\end{align*}

These calculations motivate constructing a one-step estimator through the following steps:

1. Construct estimators of all nuisance parameters using regression. For each $ g^{*}_{a} $, use the loss function in (5). The IPS weight function $ 1/\pi(X, Z, A,\tilde{T},\Delta) $ may be estimated by logistic regression. For the other nuisance parameters, one choice uses the empirical estimator of $ P(Z=0) $, parametric or superlearner regression for each $ P(A=a|X, Z=0) $, and IPS-weighted parametric or superlearner regression for each $ P(Z=0, A=a|X, S) $ and $ P(Z=1, A=0|X, S) $. Let the nuisance estimates be denoted $ \hat{\eta} $.
2. Define the plug-in estimator $ \hat{\theta}_{a,{{\tiny plug-in}}} $ as in Equation (8).
3. For observations with $ \epsilon_{S, i}=1 $, compute $ \varphi_{a}(O_{i};\hat{\eta}) $. Among these observations, regress $ \varphi_{a}(O;\hat{\eta}) $ on $ (X, Z, A,\tilde{T},\Delta) $ using the plug-in estimate of each $ \theta_{a} $. Compute the predictions from this regression for all observations, and compute $ \phi_{a}(O_{i},\hat{\eta}) $ for all observations.
4. Define the one-step estimator as
  11 \begin{align*}\hat{\theta}_{a,{{\tiny one-step}}}=\hat{\theta}_{a,{{\tiny plug-in}}}+\frac{1}{n}\sum\limits_{i=1}^{n}\phi_{a}(O_{i},\hat{\eta}).\end{align*}

For the plug-in estimator to be consistent, in the observational study each regression estimator $ \widehat{g}^{*}_{a}(X, S) $ must be consistent for $ g^{*}_{a}(X, S) $, for $ a\,=\,0,1 $, and $ \hat{\pi}(X, Z, A,\tilde{T},\Delta) $ must be consistent for $ \pi(X, Z, A,\tilde{T},\Delta) $ in both studies $ Z\,=\,0,1 $. The additional nuisance estimates must also be consistent for their respective parameters. The efficient estimator can garner improved precision over the plug-in estimator by accounting for information in the phase-one data $ (X, Z , \tilde{T},\Delta) $ that is not included for the plug-in estimator. Under A1–A8, consistent estimation of $ \pi(\cdot) $, and by selecting nuisance regression estimators that are not too data-adaptive such that all of the nuisance parameter estimators meet convergence rate conditions, both the plug-in and one-step estimators have asymptotically normal distributions.]

With complete data and a perfect surrogate, Chen and Ritzwoller (2023) proved that the EIF [their Theorem 3.1(2)], which is the same as our EIF (9) under the perfect surrogacy assumption, is unique [their Theorem 3.2(2)] and can be used to derive the semiparametric efficiency bound [their Corollary 3.1 (2). This implies that all regular and asymptotically linear estimators with the EIF as their influence function have the same asymptotic variance and attain the semiparametric efficiency bound. By adding our condition A7 regarding missing data on $ S $, their proofs apply to establish that our one-step estimator is consistent, asymptotically normal, and semiparametric efficient under regularity conditions. To handle missingness of $ Y $, the outcome piece of the EIF [first line of Equation (9)] can be modified by placing the indicator that $ Y $ is observed in the numerator and the probability of observing $ Y $ conditional on $ (A, X) $ in the denominator, yielding a consistent estimator under A8. This approach is efficient if $ Y $ has simple yes-or-no missingness but is inefficient if $ T $ can be right-censored before $ t_{0} $, because the estimator does not use the partial follow-up information.

Both estimators have the limitation of being designed to work best for an unbounded $ Y $ (ie a continuous target outcome), as they do not take into account the structural knowledge that $ Y\in\{0,1\} $. A targeted minimum loss-based estimator (eg Benkeser et al. 2018) can provide improved finite-sample performance over the one-step estimator by enforcing the structural knowledge.

#### 3.6.3. Estimation of the outcome regression/surrogate index: Engine of transport

We now discuss how to estimate the surrogate index $ g(X, S) $ in the $ Z\,=\,1 $ study. With no right-censoring during follow-up, a parametric or semiparametric model for a dichotomous outcome $ Y\,=\,I(T\leq t_{0}) $ completely observed could be used. With right-censoring, a parametric or semiparametric survival model could be applied. However, because consistent estimation of TE depends on a correctly specified model for $ g(X, S) $, it is desirable to seek flexible estimation of $ g(X, S) $. Ensemble-based super-learning (van der Laan et al. 2007) provides one approach, as considered by Price et al. (2018) for obtaining the estimated optimal surrogate (EOS) that is defined as the optimal estimator of $ g(X, S) $ by minimizing cross-validated risk.

The theoretical results of Price et al. (2018) did not consider missing data on $ S $; for our problem this is needed. One approach employs IPS weighted superlearner (Rose and van der Laan 2011), including the IPS weights in all of the individual learners that are members of the selected superlearner library. With right-censoring of $ T $, the weights can also incorporate estimates for each observed failure event the reciprocal probability of not being right-censored by their failure time (Robins and Finkelstein 2000). Another possible estimator in the right-censoring case is a debiased superlearner estimator (Wolock et al. 2024), implemented in the R package *survML* available at Charles Wolock’s GitHub page. Given the target outcome is a rare event, estimators constrained by a maximum possible event probability would be expected to provide finite-sample precision gains (Balzer and van der Laan 2013; Benkeser et al. 2018).

#### 3.6.4. Intercurrent events

Intercurrent events (ICEs) in our context are events that occur after enrollment/treatment initiation and affect either the interpretation or existence of the surrogate endpoint and/or target outcome (Saddiki and Balzer 2020). Mapping of the causal parameters to statistical estimands requires dealing explicitly with ICEs (Dahabreh et al. 2023a). For our context, relevant ICEs include (1) events occurring before the surrogate endpoint is measured that make the surrogate endpoint undefined, which would include death, or, for many applications, the target outcome; and (2) events occurring after the surrogate endpoint is measured that makes the target outcome undefined, which may include death. For some disease contexts, allowing the target outcome to include death in a composite endpoint may address a relevant question, whereas for other contexts excluding deaths may yield a more desirable interpretation. For example, excluding death would be warranted if the disease under study has very low probability of causing death (much lower than the rate of $ Y\,=\,1 $) such that most deaths are unrelated to the disease.

Studies of preventive vaccines illustrate a setting where target outcome occurrence before the surrogate endpoint is measured may be considered to render the surrogate endpoint ill-defined. For example, suppose the intent for the biomarker $ S $ is to measure an antibody response induced solely by vaccination. Occurrence of the infectious disease outcome would generate an antibody response that makes $ S $ reflect a mixture of antibodies made by vaccination and by the infection. Including such participants makes it more difficult to model $ g(X, S) $ and makes the target population a mixture of 2 quite distinct immunological groups.

Section SB discusses considerations for potentially relaxing our set-up that only includes participants who do not experience the target outcome by time $ \tau $.

### 3.7. Step 6: Quantify the uncertainty in the estimate of the statistical estimand

To meet the provisional approval bar of “reasonably likely to infer sufficient treatment efficacy,” the quantification of uncertainty in the estimation of TE should account for the spectrum of relevant uncertainty sources, including (1) sampling variability in the estimation of $ g(X, S) $ in the observational study; (2) sampling variability of $ X $ and $ S $ in the phase 3 study; (3) margin for error due to the observational and phase 3 studies having different untreated population conditional distributions of $ Y $ given $ X $ and $ S $; (4) margin for error for an imperfect surrogate; and (5) margin for error due to any simplifications that are made in the handling of ICEs.

For the plug-in estimator of TE, uncertainty sources (1) and (2) can be accounted for by sandwich variance estimation or the bootstrap if a parametric or semiparametric model is used to estimate $ g(X, S) $, including if superlearner is used with not too overly-adaptive learners. That is, under all the identifiability conditions plus correctly specified models, the sandwich variance and the bootstrap provide asymptotically correct variance estimators for $ \widehat{E}[Y(0)|Z\,=\,0] $ and $ \widehat{E}[Y(1)|Z\,=\,0] $, and hence for $ \widehat{{\rm TE}} $. The sandwich variance estimator for each $ \widehat{E}[Y(a)|Z\,=\,0] $ is derived using a stacked estimating equation (Stefanski and Boos 2002) that includes equations for each parameter in (8), most of which are nuisance parameters (Section SD). The bootstrap re-samples from both the observational and phase 3 studies.

If superlearner with highly data-adaptive learners is used, then both sandwich variance estimation and the bootstrap do not provide asymptotically correct inferences. Remedies include the highly-adaptive lasso (HAL) (Benkeser and van der Laan 2016) or use of cross-fitting in the variance estimation (eg Bickel 1982; Robins et al. 2008; Westling et al. 2024). Another option for nonparametric estimation/inference is Wolock et al. (2024). For the one-step estimator $ \hat{\theta}_{a,{{\tiny one-step}}} $, for each $ a $, under regularity conditions the variance can be consistently estimated by the empirical variance of $ \phi_{a}(O_{i},\hat{\eta}) $ across the $ n $ observations.

#### 3.7.1. Specification of the bias functions

Uncertainty sources (3) and (4) can be addressed by specifying the bias functions $ u^{UC}(X, S) $ and $ u^{CT}(X, S) $, respectively, that conservatively make estimates of TE smaller. A positive value of $ u^{UC}(X, S) $ makes both the estimates of $ E[Y(0)|Z\,=\,0] $ and $ E[Y(1)|Z\,=\,0] $ smaller, such that both negative and positive values of $ u^{UC}(X, S) $ could make TE smaller. (However, if $ E[Y(1)|Z\,=\,0] < E[Y(0)|Z\,=\,0] $, then only negative values of $ u^{UC}(X, S) $ can make TE smaller.) In contrast, $ u^{CT}(X, S) $ only affects $ E[Y(1)|Z\,=\,0] $, where a positive value of $ u^{CT}(X, S) $ makes the estimate of $ E[Y(1)|Z\,=\,0] $ larger and hence TE smaller. Therefore a conservative analysis focuses on positive values of $ u^{CT}(X, S) $. In conclusion, a conservative sensitivity analysis can be set up by defining maximum negative and maximum positive plausible values of $ u^{UC}(X, S) $, and a maximum positive plausible value for $ u^{CT}(X, S) $.

Once plausible sets are specified for the 2 bias functions, for every specific choice of $ u^{UC}(X, S) $ and $ u^{CT}(X, S) $ fixed, point estimates are obtained for $ E[Y(1)|Z\,=\,0] $, $ E[Y(0)|Z\,=\,0] $, and TE, and the variance estimation accounting for uncertainty sources (1) and (2) yields a 95% confidence interval for each of these 3 causal parameters. Jointly accounting for uncertainty sources (1)–(4) can be achieved by reporting the range of point estimates (ie ignorance interval) and the union/envelope of 95% confidence intervals for each causal parameter [ie 95% estimated uncertainty interval (Vansteelandt et al. 2006)]. The success criterion for provisional approval could be defined by minimal bars for the left endpoint of the ignorance interval for TE and the left endpoint of the 95% estimated uncertainty interval for TE, or only by the latter left endpoint. Section SE discusses approaches to specifying $ u^{UC}(X, S) $ and $ u^{CT}(X, S) $. In addition, a valuable peer-reviewer comment suggested a tipping point sensitivity analysis, which to some extent obviates the thorny challenge of pre-specifying the bias functions. Our tipping point implementation included in the R code Vignette uses constant bias functions, with 2 analyses conducted. First, with $ u^{UC}=0 $, calculate the largest magnitude positive value of $ u^{CT} $ at which the lower 95% confidence limit for TE exceeds 0.30. Second, with $ u^{CT}=0 $, calculate the largest magnitude negative value of $ u^{UC} $ at which the lower 95% confidence limit for TE exceeds 0.30, where only negative $ u^{UC} $ values need to be considered because $ E[Y(1)|Z\,=\,0] < E[Y(0)|Z\,=\,0] $ is supported by meeting the success criterion (see Step 6).

These analyses quantify how much isolated departure from perfect (zero-bias) versions of Surrogacy A6 and Comparability A4, respectively, overturns meeting the success criterion defined by the lower 95% confidence limit for TE lying above 0.30.

### 3.8. Step 7: Compare feasible analytic designs (Steps 1–6) using simulation studies

The statistical analysis plan for the phase 3 study needs to be completed before availability of the phase 3 data. To help develop this plan, it could be useful to conduct outcome-blind simulations of the observational and phase 3 studies to fine-tune the choices that must be made, such as on: (i) how to estimate $ g(X, S) $ including which variables $ X $ and $ S $ to include; (ii) how to specify the bias functions $ u^{UC}(X, S) $ and $ u^{CT}(X, S) $; and (iii) how to ensure that EUIs for the $ TE $ causal parameter of interest adequately capture uncertainty in both the observational study and phase 3 study. Given that this manuscript develops new estimation and inference procedures (plug-in, one-step) that we have not yet vetted, we conduct a simulation study of standard performance metrics (bias, power, validity of inferences), all mimicking anticipated forthcoming real data from GBS vaccine development.

## 4. CASE STUDY: APPLICATION TO GROUP B STREPTOCOCCUS VACCINATION

For the past several years the GBS vaccine field has been pursuing a provisional approval pathway based on antibody markers measured in infant cord blood [IgG antibody levels to capsular or Alpha proteins and Opsonophagocytosis Killing Assay readouts] that have been shown to strongly inversely correlate with IGbsD in natural history studies (eg Madhi et al. 2021, 2023; Dangor et al. 2023). Vaccine developers are currently pursuing a development pathway that learns about a surrogate endpoint from multiple observational studies and then estimates vaccine efficacy in a phase 3 target trial based on this surrogate. The ongoing observational studies are the COP01-WITS/GBS Alpha study in South Africa, the COP02-SGUL/iGBS3 study in the United Kingdom, and the EDCTP-sponsored PREPARE study in Denmark, France, Italy, Malawi, the Netherlands, South Africa, Uganda, United Kingdom, United States, which are similar to 2 recently published studies in South Africa (Madhi et al. 2021, 2023). Each study collects cord blood samples and/or acute-illness samples from infant IGbsD cases from 0 to 90 days of age and cord blood samples from non-cases/controls, enabling analyses to assess the association of cord-blood antibody markers with IGbsD.

Phase 3 GBS vaccine trials in planning randomize pregnant mothers to receive 2 vaccine or placebo doses starting in the third trimester, with cord blood collected from all infants for potential measurement of the set of antibody markers $ S $ (therefore, time $ \tau $ is the birth/delivery visit). The antibody markers will be measured from a case-cohort sample of live-born infants of vaccinated and placebo mothers. The visit schedule and sampling of cord blood is harmonized across the multiple observational studies and the phase 3 study, and the same antibody markers are measured using the same instruments and protocol. The primary analysis estimates vaccine efficacy (VE) against IGbsD for the phase 3 sub-population of live-born infants based on the surrogate index that is learned (ie an estimate of $ g(X, S) $) from data analysis of the observational studies pooled.

Section SF details Steps 1–6 of the Provisional Approval Causal Roadmap for the GBS application. We describe Step 7 next because it provides finite-sample performance characteristics of the proposed estimators.

### 4.1. Step 7: Simulation study to plan for VE estimation in the GBS vaccine phase 3 study

We generate data for mother-infant (live born) dyads in the sero-epidemiological observational study or the phase 3 placebo-controlled vaccine trial. We design simulation conditions in the observational study to roughly match published GBS characteristics. In particular, we specify probability of IGbsD by 90 days at 0.005 (Vekemans et al. 2019b) and geometric mean cord-blood IgG concentration of 0.01 and 0.04 in IGbsD cases and controls (observed free of IGbsD through 90 days of age), respectively (Dangor et al. 2023). We consider $ X=(X_{1},X_{2},X_{3}) $ with $ X_{1} $ and $ X_{2} $ 2 known prognostic risk factors for IGbsD: $ X_{1} $ the indicator of gestational age <37 weeks, and $ X_{2} $ maternal age in years where younger age is a risk factor. We also add a continuous noise variable $ X_{3} $ unrelated to outcome (an accidentally adjusted for variable). For the phase 3 study, the same variables are simulated. Section SG details the simulation conditions.

Three simulation studies of the plug-in and one-step estimators are conducted. First, data are simulated with both bias functions equal to zero, and we evaluate estimator bias, variance, and coverage of 95% confidence intervals for $ E[Y(a)|Z\,=\,0] $ ($ a\,=\,0,1 $) and VE when conducting the analysis with the bias functions set to zero. The purpose of this simulation study is to verify correct properties of estimator performance under known conditions. Secondly, we conduct the analysis using specified bias functions to make inferences on VE conservative, with purpose to evaluate power to meet the success criterion (defined as the 95% EUI for VE $ \geq 0.3 $) for realistic methods’ implementations that requires non-zero bias functions. Our third simulation study considers the plug-in estimator with an incorrectly specified parametric model, to evaluate the flexibility advantage of the one-step estimator.

#### 4.1.1. Simulation study 1: Ideal conditions with zero bias functions and no sensitivity analysis

With $ u^{UC}(X, S) $ and $ u^{CT}(X, S) $ set to zero, we simulate data for 39,000 participants in the sero-epidemiological study ($ Z\,=\,1, A\,=\,0 $) and 6,200 participants in the phase 3 vaccine trial ($ Z\,=\,0 $), with 3,100 each randomized to vaccine ($ A\,=\,1 $) and placebo ($ A\,=\,0 $). We fix the following conditions to be the same in the 2 studies: (i) $ X=(X_{1},X_{2},X_{3}) $ where $ X_{1}|Z\sim\mathrm{Bernoulli}(0.05) $, $ X_{2}|Z\sim\mathrm{Uniform}(18,40) $, and $ X_{3}|Z\sim\mathrm{Normal}(0,1) $; (ii) $ S|X, A\,=\,0, Z\sim\mathrm{Normal}(-1.45,0.00225) $; and (iii) $ Y|X, A\,=\,0, Z, S\sim $ Bernoulli with $ \mathrm{logit}P(Y\,=\,1|A\,=\,0, Z, S, X_{1},X_{2},X_{3})=-17.1-8.2S\,+\,0.69X_{1}-0.03X_{2} $. For simplicity we assume $ Y\,=\,I(T\leq t_{0}) $ is always observed; i.e., no right-censoring by $ t_{0} $ = 90 days.

To induce $ u^{CT}(X, S)=0 $, we set the conditional distribution function of $ Y $ equal for the vaccine and placebo arms: $ P(Y\,=\,1|A\,=\,1, Z\,=\,0, S, X_{1},X_{2},X_{3})=P(Y\,=\,1|A\,=\,0, Z\,=\,0, S, X_{1},X_{2},X_{3}) $. To generate VE $ =\{0,0.5,0.9\} $, we manipulate the distribution of the biomarker in the vaccine arm, $ S|A\,=\,1, Z\,=\,0 $. The value VE = 0, 0.5, 0.9 is implied by setting $ S|A\,=\,1, Z\,=\,0\sim\mathrm{Normal}(-1.45,0.0225),\mathrm{Normal}(-1.296,0.04) $, and $ \mathrm{Normal}(-1.08,0.0441) $, respectively.

Finally, we set a case-control sampling design for measuring $ S $ in cord blood in the observational study as follows: we sample $ S $ from all IGbsD cases and a simple random sample of controls with the number set to 5 times the number of cases. In the phase 3 trial, we sample $ S $ from a simple random sample of 100, 250, or 500 infant participants in each randomization arm.

For estimation and inference with the plug-in estimator, we first estimate $ g(X, S) $ with a correctly specified IPS-weighted logistic regression of $ Y $ on $ (X, S) $ including participants with $ S $ measured in the observational study. The IPS weight $ \pi(X, Z, A,\tilde{T},\Delta) $ is set to the true sampling weights: 1 for cases and $ n_{controls}/5n_{cases} $ for the controls, where $ n_{cases} $ is the number of participants with $ Y\,=\,1 $ and $ n_{controls} $ is the number of participants with $ Y\,=\,0 $. The expected number of observational study cases with $ S $ measured is 0.005*39,000 = 195. To obtain an estimate for the expectation in the summand of (8), we then regress the fitted values $ \hat{g}(X, Z\,=\,1, S) $ on $ X $, in the phase 3 study ($ Z\,=\,0 $) in each arm using IPS-weighted linear regression.

We then use the plug-in estimator Equation (8) to calculate $ \hat{E}[Y(0)|Z\,=\,0] $ and $ \hat{E}[Y(1)|Z\,=\,0] $, yielding $ \log(1-\widehat{{\rm VE}})=\log\{\hat{E}[Y(1)|Z\,=\,0]/\hat{E}[Y(0)|Z\,=\,0]\} $. We calculate bootstrap and sandwich variances for $ \hat{E}[Y(0)|Z\,=\,0] $, $ \hat{E}[Y(1)|Z\,=\,0] $, and $ \log(1-\widehat{{\rm VE}}) $. The bootstrap re-samples cases and controls separately in the observational data set, and vaccine and placebo recipients separately in the phase 3 data set. From these variance estimates, we obtain Wald confidence intervals. To estimate $ {\rm VE} $ and its corresponding confidence interval, we transform the point estimate and symmetric confidence interval limits around $ \log(1-\widehat{{\rm VE}}) $.

For the one-step estimator, we first estimate the nuisance functions: $ g(X, S) $, $ E[\,\widehat{g}_{a}(X, S)|X, Z\,=\,0, A_{i}=a] $, $ P(Z\,=\,0) $, $ \pi(X, Z\,=\,1, A\,=\,0 , \tilde{T},\Delta)=P(\epsilon_{S}=1|X, Z\,=\,1, A\,=\,0, Y) $ (with no censoring), $ P(A\,=\,a|X, Z\,=\,0) $, $ P(Z\,=\,0, A\,=\,a|X, S) $, and $ P(Z\,=\,1, A\,=\,0|X, S) $:

• For both $ g(X, S) $ and $ E[\,\widehat{g}_{a}(X, S)|X, Z=0, A_{i}=a] $, we use IPS-weighted superlearner with the true sampling weights.
• Use the empirical estimator $ \hat{P}(Z=0)=\frac{1}{n}\sum_{i=1}^{n}I(Z_{i}=0) $.
• For $ P(A=a|X, Z=0) $, we use the true randomization probability from the Phase 3 study.
• We estimate $ P(\epsilon_{S}=1|Z, A, X) $ with logistic regression, by regressing $ \epsilon_{S} $ on $ A, X $ separately for each study $ Z=0,1 $, to obtain $ \hat{P}(\epsilon_{S}=1|Z=0, A, X) $ and $ \hat{P}(\epsilon_{S}=1|Z=1, A, X) $.
• We estimate $ P(Z=0, A=a|X, S) $ by first creating a dummy variable that takes the value of 1 if $ Z=0, A=a $, and then perform IPS-weighted superlearner regression, regressing this dummy variable on $ X, S $, using as sampling weights the inverse of $ \hat{P}(\epsilon_{S}=1|Z, A, X) $.
• We estimate $ P(Z=1, A=0|X, S) $ by first noting that $ \hat{P}(Z=1, A=0|X, S)=\hat{P}(Z=1|X, S) $. We then regress $ Z $ on $ X, S $ with IPS-weighted superlearner, using as sampling weights the inverse of $ \hat{P}(\epsilon_{S}=1|Z, A, X) $.
• All superlearner estimations include generalized additive models, generalized linear models, and means as candidate learners.

After estimating the nuisance functions, we then follow Steps 2–4. of the section on the nonparametric efficient one-step estimator, and calculate the one-step estimator using Equation (11). For $ \hat{E}[Y(0)|Z\,=\,0] $ and $ \hat{E}[Y(1)|Z\,=\,0] $, we estimate the variance by $ 1/n $ times the sample variance of the sum of the influence function contributions $ \phi_{a}(O_{i},\hat{\eta}) $. We obtain the variance estimate for $ \log(1-\widehat{VE}) $ using the delta method, before transforming estimates and symmetric 95% confidence interval limits to the VE scale.

Across 800 simulations, we report bias and confidence interval coverage for $ \hat{E}[Y(0)|Z\,=\,0] $, $ \hat{E}[Y(1)|Z\,=\,0] $, and $ \widehat{VE} $ for both estimators. Additionally, we report variance estimates and Monte Carlo empirical standard errors for $ \hat{E}[Y(0)|Z\,=\,0] $, $ \hat{E}[Y(1)|Z\,=\,0] $, and $ \log(1-\widehat{{\rm VE}}) $.

#### 4.1.2. Simulation study 1: Results

**Fig. 3.**
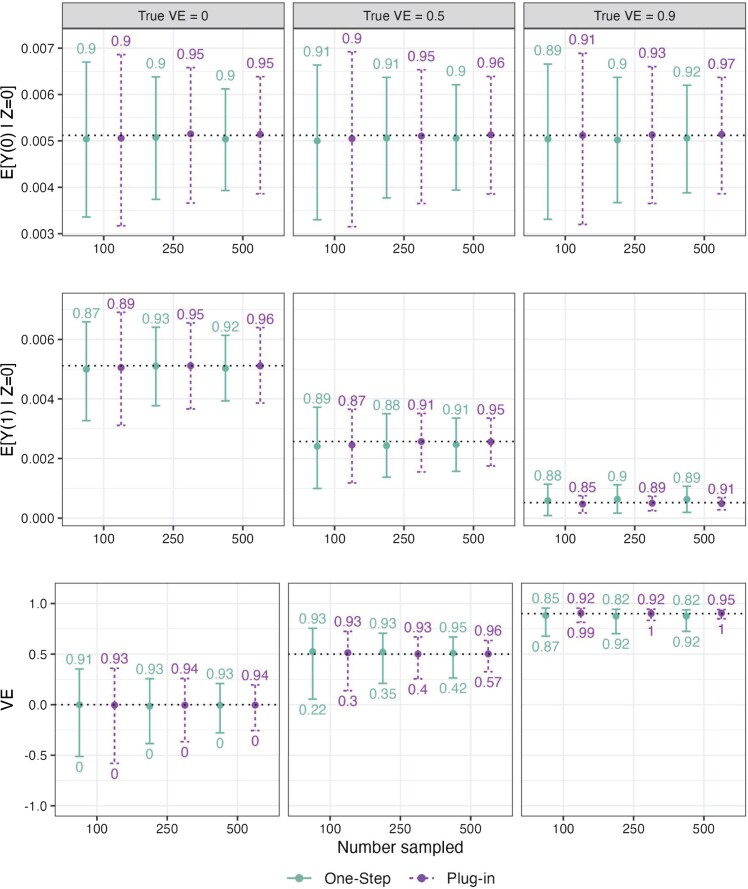
Results for Simulation Study 1 across true VE = 0, 0.5, and 0.9 for the one-step and plug-in estimators. The top, middle, and bottom panels display results for estimating $ E[Y(0)|Z\,=\,0] $, $ E[Y(1)|Z\,=\,0] $, and VE, respectively. Each graph depicts results across 100, 250, and 500 with $ S $ data in each of the vaccine and placebo arms of the phase 3 trial $ Z\,=\,0 $. Points represent the median estimate across simulations, and error bars display the median lower and upper 95% confidence interval bounds (derived from sandwich standard errors for the plug-in estimator and from influence-function standard errors for the one-step estimator). Dotted horizontal lines are placed at the true value. The numbers above each error bar display the 95% confidence interval coverage, and the numbers below the error bar in the VE panel show power to meet the success criterion (defined as 95% EUI for VE $ \geq 0.3 $).

For all 3 Simulation Study 1 settings and both estimators, we observed low bias, with slightly smaller bias observed with the plug-in estimator (Fig. 3, Table S1). For both estimators, standard errors generally decreased and confidence interval coverage increased with increasing numbers sampled. For the plug-in estimator, approximately nominal coverage of 95% confidence intervals was obtained for $ E[Y(0)|Z\,=\,0] $, $ E[Y(1)|Z\,=\,0] $, and VE in the setting where 500 participants were sampled for each treatment arm of the $ Z\,=\,0 $ study. However, with the one-step estimator, we observed confidence interval under-coverage for both $ \hat{E}[Y(1)|Z\,=\,0] $ and $ \log(1-\widehat{{\rm VE}}) $ in the true VE $ =0.9 $ scenario, across all numbers sampled. In this scenario, the support of $ S $ in the vaccine arm of the phase 3 trial ($ Z\,=\,0, A\,=\,1 $) lies partially outside of the range of the support of the observational trial data ($ Z\,=\,1 $) used to estimate $ g(X, S) $. While the plug-in estimator using correctly specified logistic regression performs well extrapolating these fitted values, the superlearner nuisance estimation within the one-step estimator performs worse with this extrapolation, leading to poorer estimation of $ E[Y(1)|Z\,=\,0] $.

For the plug-in estimator, we observed small discrepancies between the bootstrap and sandwich standard errors, where for VE the bootstrap gave slightly larger estimates. Empirical variances tended to be higher than both estimated variances, with larger differences in settings where fewer participants were sampled and with the highest true VE. We believe this is due to the rare-event setting with a limited number of $ Y\,=\,1 $ outcomes. Additional simulations increasing the failure rate by about 3-fold showed closer agreement between the bootstrap, sandwich, and Monte Carlo standard errors (Section SG).

#### 4.1.3. Simulation study 2: Non-zero bias functions

The purpose of the second simulation study is to estimate power of the different methods to meet the provisional approval success criterion, defined as the 95% EUI for VE lying above 0.30, under a realistic implementation of the methods. Realistic implementation means carrying out the analysis building in conservative margin via specification of the bias functions. Because for GBS the observational and phase 3 study populations are similar, we set $ u^{UC}=0 $ and focus on the $ u^{CT} $ bias function that specifies how much the surrogate endpoint $ S $ departs from perfection. In particular, we use a specified lower bound for the proportion of the treatment effect on the target outcome explained by $ S $ (PTE) (Freedman et al. 1992) as a way to define a worst-case constant bias function value $ u^{CT} $, where Lin et al. (1997) suggested PTE at least 0.5 as a minimum requirement for a reasonable surrogate. Specifically, using formula (E.1) in Section SE, with TE = 0.7, $ P(Y(0)=1|Z\,=\,0)=0.005 $, and PTE($ X $) set to 0.67, 0.83, or 1.0, the resulting bound for $ u^{CT} $ is 0.0012, 0.00060, or 0, respectively. Data are simulated as in Simulation Study 1, with 250 sampled for $ S $ measurement in each treatment arm of the $ Z\,=\,0 $ trial. We simulate data under true $ u^{CT}=0 $, to study the power of the methods when they conservatively assume more bias than is present.

#### 4.1.4. Simulation study 2: Results

**Fig. 4.**
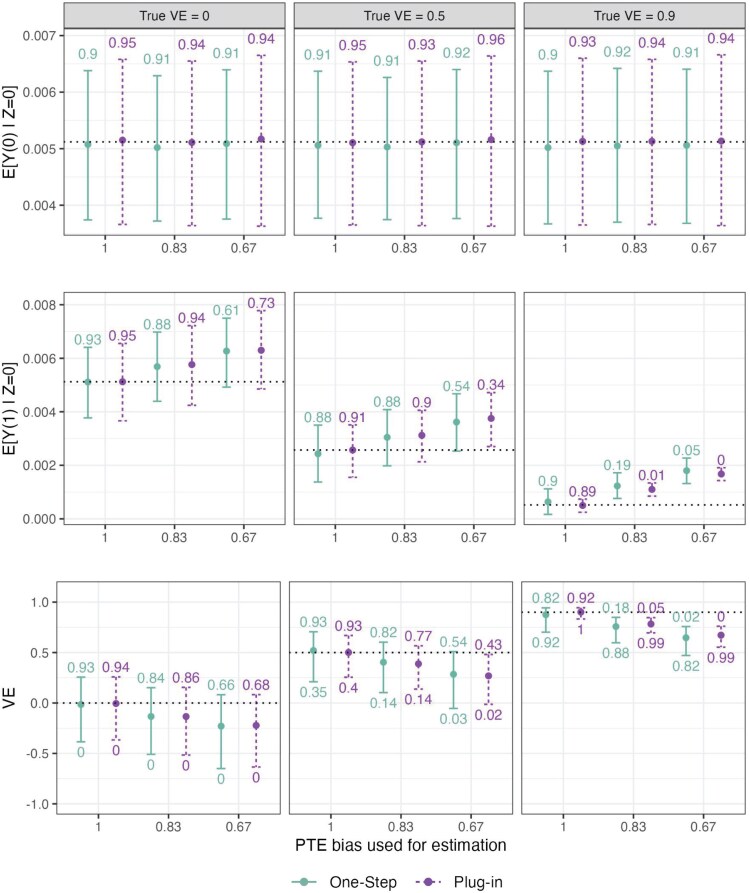
Results for Simulation Study 2 across true VE = 0, 0.5, and 0.9 for the one-step and plug-in estimators. The top, middle, and bottom panels display results for estimating $ E[Y(0)|Z\,=\,0] $, $ E[Y(1)|Z\,=\,0] $, and VE, respectively. The $ S $ data are sampled from 250 participants in each treatment arm of the phase 3 study $ Z\,=\,0 $. Each graph depicts results across different bias functions $ u^{CT}(X, S)=u^{CT} $ used for estimation corresponding to PTE = 1, 0.83, and 0.67. Points represent the median estimate across all simulations, and error bars display the median lower and upper 95% confidence interval bounds (derived from sandwich standard errors for the plug-in estimator and from influence-function standard errors for the one-step estimator). Dotted horizontal lines are placed at the true value. The numbers above each error bar display the confidence interval coverage, and the numbers below the error bar in the VE panel show power to meet the success criterion (defined as 95% EUI for VE $ \geq 0.3 $).

Results for Simulation Study 2 are shown in Fig. 4 and Table S2. With both estimators, we observe over-estimation of $ E[Y(1)|Z\,=\,0] $ and underestimation of VE as we increase the $ u^{CT} $ bias used for estimation, while estimation of $ E[Y(0)|Z\,=\,0] $ is unaffected. This is as expected, since the purpose of including the $ u^{CT} $ bias function is to conservatively make estimates of $ E[Y(1)|Z\,=\,0] $ larger and estimates of VE smaller. The estimator $ \widehat{E}[Y(0)|Z\,=\,0] $ is unchanged because we do not vary $ u^{UC} $ bias. For the true VE = 0.5 scenario, with the plug-in estimator, empirical provisional success probabilities were 40%, 14%, and 2%, for increasing specified bias based on setting PTE $ =1,0.83 $, and 0.67, respectively. With the one-step estimator, success probabilities were 35%, 14%, and 3%. For the true VE = 0.9 scenario, empirical success probabilities for the plug-in estimator were 100%, 99%, and 99% for the 3 bias scenarios compared to 92%, 88%, and 82% for the one-step estimator.

#### 4.1.5. Simulation study 3: Advantage of the nonparametric efficient one-step estimator

Third, we study the scenario where the model used to estimate $ g(X, S) $ in the plug-in estimator is incorrectly specified. In this setting the one-step estimator has the advantage of utilizing flexible nuisance function estimation. We generate the data as follows for both studies $ Z=\{0,1\} $: (i) $ X $ simulated as in Simulation Studies 1 and 2; (ii) $ S|X, A\,=\,0, Z\sim\mathrm{Normal}(-1.45,0.09) $; (iii) $ Y|X, A, Z, S\sim $ Bernoulli with $ \mathrm{logit}\{(1/0.007)*P(Y\,=\,1|A\,=\,0, Z, S, X_{1},X_{2},X_{3})\}=-8.6-1.5S\,+\,4.4S^{2}+ 0.69X_{1}-0.03X_{2} $; (iv) True bias functions $ u^{UC}(X, S) $ and $ u^{CT}(X, S) $ are zero. Note that for (iii) a logistic regression model is mis-specified.

The VE values 0, 0.5, and 0.9 were examined, implied by setting $ S|A\,=\,1, Z\,=\,0\sim\mathrm{Normal}(-1.45,0.09),\mathrm{Normal}(-1.22,0.0361) $, and $ \mathrm{Normal}(-1.045,0.01) $, respectively. Simulation Study 1 specified the distribution of $ S $ substantially different between the observational and phase 3 studies, generating a challenging setting for the one-step estimator as it’s flexible estimation cannot extrapolate outside the support of $ S $. To create a more favorable setting for the one-step estimator, for Simulation Study 3 the distributions of $ S $ were specified to be more overlapped between the 2 studies. Simulation Study 3 uses the same sample size and $ S $ sampling structure as in Simulation Study 1. Moreover, in the $ Z\,=\,0 $ study, we sample 250 participants for each arm $ A\,=\,0,1 $ for measurement of $ S $.

#### 4.1.6. Simulation study 3: Results

**Fig. 5.**
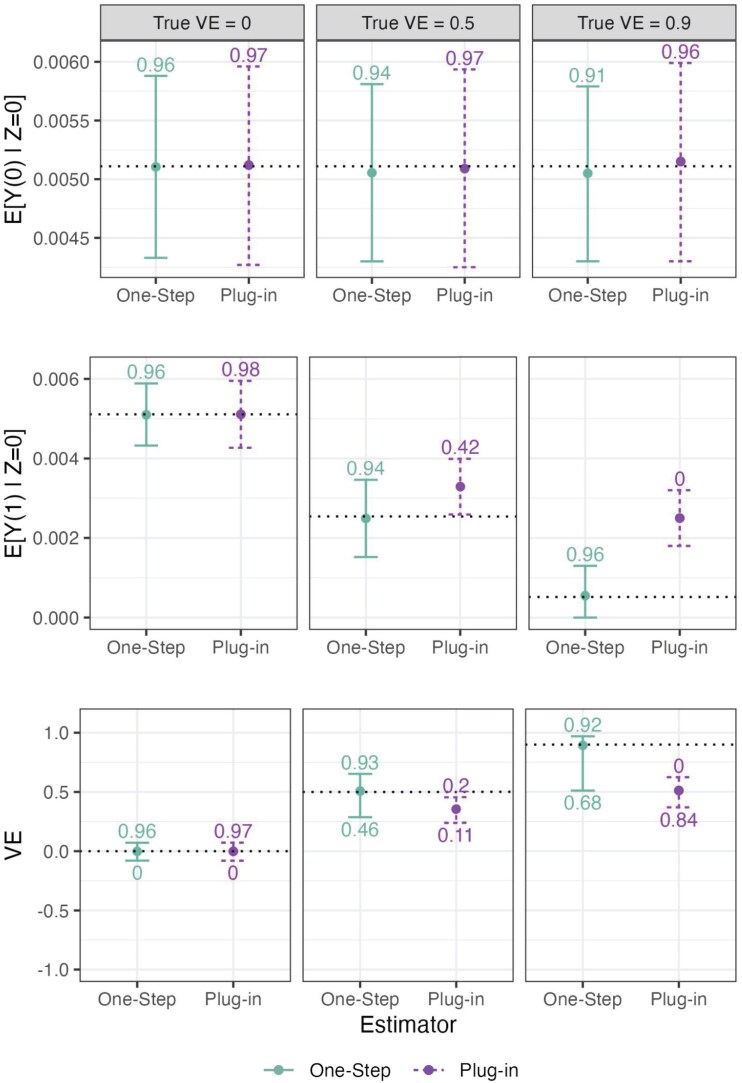
Results for Simulation Study 3 across true VE = 0, 0.5, and 0.9 for the one-step and plug-in estimators. The top, middle, and bottom panels display results for estimating $ E[Y(0)|Z\,=\,0] $, $ E[Y(1)|Z\,=\,0] $, and VE, respectively. The $ S $ data are sampled from 250 participants in each of the vaccine and placebo arms of the phase 3 trial $ Z\,=\,0 $. Points represent the median estimate across all simulations, and error bars display the median lower and upper 95% confidence interval bounds (derived from sandwich standard errors for the plug-in estimator and from influence-function standard errors for the one-step estimator). Dotted horizontal lines are placed at the true value. The numbers above each error bar display the confidence interval coverage, and the numbers below the error bar in the VE panel show power to meet the success criterion (defined as 95% EUI for VE $ \geq 0.3 $).

Results are shown in Fig. 5 and Table S3. While both estimators of $ E[Y(0)|Z\,=\,0] $ perform well, the plug-in estimator of $ E[Y(1)|Z\,=\,0] $ is highly biased with poor confidence interval coverage when true VE = 0.5 or 0.9. In contrast, the one-step estimator performs with minimal bias and approximately nominal confidence interval coverage for $ E[Y(1)|Z\,=\,0] $ and $ VE $ in these settings. This is not surprising, as the one-step estimator is able to correctly estimate the nuisance functions with flexible superlearner estimation, while the plug-in estimator relies on an incorrectly specified parametric model.

##### 4.1.7. Vignette application to a single data set

The real data from both the observational and phase 3 GBS vaccine studies are not yet available for data analysis. For facilitating reproducible research and application of the methods to data sets, Section SG.3 provides a vignette for a single simulated data set, which is implemented in the R code.

## 5. DISCUSSION

Motivated and illustrated by contemporary Group B Streptococcus vaccine development, this article considers a context that occurs for many rare diseases, where there are promising preventive interventions and a promising surrogate endpoint(s) that is strongly associated with a target disease outcome (learned from observational studies), yet it has proven elusive to conduct a pivotal phase 3 trial that could provide direct evidence demonstrating a beneficial intervention effect to prevent the target outcome. This article applies the Causal Roadmap rubric to define a surrogate-based Provisional Approval Causal Roadmap, detailing a recipe for defining target parameters, identifiability assumptions, estimators, and optimization of those estimators. This approach combines prospective observational study data, which include data on the surrogate and the target outcome and can be used to estimate their relationship, with a phase 3 randomized, treatment-vs.-control surrogate endpoint study that collects the same data, but, because by design it is massively under-powered to assess the treatment effect (TE) on the target outcome, the goal is conservative inference for treatment efficacy (TE) based on the surrogate to support provisional approval. Indeed, it is this under-powering that led to our approach to treat the Untreated-to-Control and Control-to-Treated bias functions as fixed sensitivity parameters instead of estimating them from the data. Our approach accounts for 2-phase sampling of the surrogate in both studies and for right-censoring of the target outcome in the observational study. Given that for very rare diseases the observational study or studies will need to be huge to adequately learn the outcome regression/surrogate index, a disease-specific registry may be a particularly valuable source. Applicable registries would need to include sample collection enabling biomarker measurement and ample follow-up for disease outcomes.

A potential limitation of our approach is the specification of the bias functions as additive differences in 2 conditional risks [Equations (1) and (2)] that are constrained by 0-1 probability bounds, requiring care to avoid accidentally specifying the bias functions incompatibly with these bounds. Future work could eliminate this issue by altering (1) and (2) by applying a logit transformation to each conditional risk, leading to new estimators including a different efficient influence function.

One of the needed assumptions, A3, requires a common support of the surrogate endpoint in the treatment and control arms of the phase 3 trial. However, if the new treatment/intervention is highly promising it may induce higher levels of the surrogate than attained in any control arm participants. To address this kind of challenge, one simple approach, consistent with the objective of the provisional approval paradigm to seek conservative/lower bound inference about TE, would truncate surrogate values at the maximal observed value of control arm participants.

The transport/bridging approach considered here separates bridging into 2 parts: Untreated-to-Control-transport from the observational study (of untreated individuals) to the control/placebo arm of the phase 3 study, and Control-to-Treated-transport within the phase 3 study. While we have suggested this 2-part approach has advantage of aiding transparency of assumptions and study design, a potential drawback is the use of 2 sets of bridging weights that could increase variability and elevate the risk of unstable inference. An alternative approach would use only one set of weights (eg Chattopadhyay et al. 2024).

## 6. SOFTWARE

The R code used for the simulation studies, and for analysis of a single simulated data set mimicking the anticipated forthcoming real GBS data, is available at https:$ // $github.com$ / $jpspeng$ / $gbsmssims

## Supplementary Material

kxaf018_Supplementary_Data

## Supplementary material

Section SA compares the present manuscript methods to Athey et al. (2024), Section SB discusses how the methods may address target outcome occurrence before the surrogate is measured, Section SC details the mapping of the causal parameters to statistical estimands, Section SD details sandwich variance estimation for the plug-in estimator, Section SE discusses specification of the 2 bias functions, Section SF applies Steps 1–6 of the Provisional Approval Causal Roadmap to the GBS case study, and Section SG elaborates the simulation study design and provides additional simulation study results.
