# Supplementary material for: A surrogate endpoint-based provisional approval causal roadmap, illustrated by vaccine development
Source: Biostatistics. 2025 Jun 22;26(1):kxaf018. doi: 10.1093/biostatistics/kxaf018 (PMC12205950; doi:10.1093/biostatistics/kxaf018)
Supplement: kxaf018_Supplementary_Data [file kxaf018_supplementary_data.pdf]

Biostatistics (2025), **0**, 0, *pp.* 1–**??**
doi:10.1093/biostatistics/output

Supplementary Material For "A Surrogate Endpoint Based Provisional Approval Causal Roadmap, Illustrated by Vaccine Development" by Gilbert et al.

A. Comparison of the Present Work to Athey et al. (2024)

We detail differences between the present manuscript and Athey *and others* (2024) in their providing general statistical methodology for the same or similar transportability objective.

1. The two articles use similar notation except our study indicator Z is their $\mathcal{P}$ and our binary treatment A is their W. In the following notes we use the notation of the present article.

2. The two articles consider the same target causal parameter of interest − TE − a contrast in $E[Y(1)|Z = 0]$ vs. $E[Y(0)|Z = 0]$. This work develops all elements allowing for a general contrast function $h(x, y)$ satisfying $h(x, y) = 0$ if and only if $x = y$, given that a multiplicative contrast such as $h(x, y) = \log(E[Y(1)|Z = 0], E[Y(0)|Z = 0])$ or $h(x, y) = 1 - E[Y(1)|Z = 0]/E[Y(0)|Z = 0]$ is needed for our provisional approval application, whereas Athey et al. restrict attention to an additive difference contrast $h(x, y) = E[Y(1)|Z = 0] - E[Y(0)|Z = 0]$. The Athey et al. results could be readily generalized to handle a general contrast.

3. This work supposes all observational study participants have treatment level $A = 0$ known whereas Athey et al. assume A is missing/unknown. An implication is Athey et al.'s Assump-

© The Author 2025. Published by Oxford University Press. All rights reserved. For permissions, please e-mail: journals.permissions@oup.com

tion 1 of a single random sample is different from our set-up. Under both the Comparability Assumption and the Surrogacy Assumption (perfect surrogate version) noted below, this difference does not affect the identifiability results nor the estimators, such that results of the two articles are equivalent in this case. Under violations of either of these assumptions (the focus for our provisional approval objective), the identifiability results and hence also the estimators are different for the two articles.

4. Related to the previous point, both articles provide a nonparametric efficient influence function for use in estimation, which are equal under both the Comparability Assumption and Surrogacy Assumption (perfect version) and differ otherwise due to the different set-up.

5. Both articles use the "optimal surrogate" (our nomenclature from Price *and others* (2018)) or equivalently "surrogate index" (Athey et al. nomenclature) as a central ingredient of the results: $E[Y|X, Z = 0, A = a, S]$. Indeed, a significant idea common to both articles is to make use of a conditional regression that can depend on multivariable S, an idea that we previously championed in Price *and others* (2018) and picked up in this work. Athey et al. provide a valuable summary of the benefits of multiple surrogates in their Section 3.2.3.

6. Both articles assume that in the experimental study $Z = 0$, treatment assignment A is unconfounded (strong ignorability), equivalently the $Z = 0$ study is randomized within levels of baseline characteristics X. This assumption is A2 in this work and Assumption 2 in Athey et al.

7. Both articles make a Comparability Assumption, in different ways. Our assumption A4 equates two conditional means offset by the Untreated-to-Control transport bias function $u^{UC}(X, S)$, and Athey et al.'s Assumption 4 expresses conditional independence.

8. Both articles use a Surrogacy Assumption (a Prentice valid surrogate: our assumption A6; Athey et al. Assumption 3) as a key assumption for the results, with difference that

our assumption is expressed in terms of equal conditional means and the Athey et al. assumption is expressed as conditional independence. For identifiability of the target causal parameter of interest, the assumption of equal conditional means suffices, with conditional independence not necessary; we speculate that it was not Athey et al.'s goal to define minimally sufficient identifiability assumptions as they had other reasons to prefer to use the stronger conditional independence assumption. In addition, the present article does not state the Surrogacy Assumption apart from a bias function given that for the provisional approval application the data analysis prioritizes scenarios with a non-zero bias function.

9. Elaborating on the last point, the two articles make a different "style choice" regarding how to include bias functions in relation to the key Surrogacy Assumption and Comparability Assumption. Athey et al. introduces these key assumptions under the ideal case that both assumptions hold, and provide results expressing the bias that results from deviations from these assumptions. The present work includes the bias functions as fixed and user-specified functions directly in the identifiability assumptions. Our provisional approval application drove this choice, where for this application data analysis assuming bias is most germane based on our understanding that regulators will generally require explicit conservative inference to provide sufficient evidence undergirding a provisional approval decision, where the pre-specified success benchmark in the statistical analysis plan would include non-zero bias functions.

10. Athey et al. included three representations of the statistical estimand of interest [their equation (4.1) and our equation (4)] using a surrogate index, a surrogate score [their Definition 2: surrogate score $P(A = 1|S = s, X = x, Z = 0)$], or both, with utility that each representation indicates different parameters that require estimation and for some applications one or another representation may be more advantageous. These representations are equations (4.2), (4.3), (4.4) in Athey et al. Our article focuses on a representation most similar to

(4.2), and in the special case that the Comparability Assumption and the Surrogacy Assumption (perfect version) hold, it is straightforward to write our statistical estimand in any of the three representations expressed by Athey et al.

11. This work emphasizes the fact that the surrogates S are measured via a two-phase sampling design in both studies, developing all results accounting for this ubiquitous data reality for the provisional approval application. Athey et al. considers complete data on the surrogates S in the observational study; the results of Athey et al. could be extended to account for two-phase sampling of S under a missing at random assumption, where there are multiple ways that the extensions could account for the missing data on S.

12. This work considered the issues of the target outcome $Y = I(T \leqslant t_0)$ being subject to right-censoring and considered Intercurrent events (ICEs).

B. Violation of the Simplifying Assumption that All Enrolled Participants in Both Studies Are Free of the Target Outcome through the Visit at τ for Surrogate Measurement

Let $Y^{0-\tau}$ be the indicator of failure after enrollment by time τ by which the surrogates are measured, and let $Y^{0-\tau}(a)$ be this potential outcome indicator under assignment a, for $a = 0, 1$. Our development made the simplifying assumption that all enrolled participants in both studies did not experience the target outcome by the visit τ at which intermediate outcomes S are measurable, i.e., $Y_i^{0-\tau} = 0$ for all i. In practice this is likely violated. We also made the simplifying assumption of no loss to follow-up before τ. To address this second issue, the methods are valid when applied only including participants observed to reach time τ, by adding a random censoring assumption conditional on (X, Z, A).

To address the occurrence of target outcome events before τ, note that for applications

that exclude participants with early failure $Y^{0-\tau} = 1$, a TE target parameter that contrasts $E[Y(1)|Z = 0, Y^{0-\tau}(1) = 0]$ and $E[Y(0)|Z = 0, Y^{0-\tau}(0) = 0]$ is not a causal parameter due to different conditioning sets. One approach to recovering a causal parameter re-defines TE as a contrast of $E[Y(1)|Z = 0, Y^{0-\tau}(1) = Y^{0-\tau}(0) = 0]$ and $E[Y(0)|Z = 0, Y^{0-\tau}(1) = Y^{0-\tau}(0) = 0]$, which measures efficacy in the "always-survivors" principal stratum of participants that are free of the target outcome by time τ under both treatment assignments. This approach has been commonly used for preventive vaccines (e.g., Gilbert *and others* (2020)). To identify this causal TE parameter, one approach makes the "equal early clinical risk" (EECR) assumption of no individual-level causal treatment effects on Y by τ $[P(Y^{0-\tau}(1) = Y^{0-\tau}(0)) = 1]$. Under this assumption a data analysis that simply excludes all early failure events works, as in this case both causal parameters equal the identified parameters:

$$E[Y(a)|Z = 0, Y^{0-\tau}(1) = Y^{0-\tau}(0) = 0] = E[Y(a)|Z = 0, Y^{0-\tau}(a) = 0],$$

for $a = 0, 1$.

For applications where it is not appropriate to assume EECR, an alternative assumption that achieves the same objective as EECR is that both of the following conditions hold with probability one:

1. $Y(1)$ and $Y^{0-\tau}(0)$ are independent conditional on $(X, S(1))$, $Y^{0-\tau}(1) = 0$ and $A = 1$;
2. $Y(0)$ and $Y^{0-\tau}(1)$ are independent conditional on $(X, S(0))$, $Y^{0-\tau}(0) = 0$ and $A = 0$,

where recall the time origin for $Y(a) := I(T(a) \leqslant t_0)$ is τ and $Y^{0-\tau}(a)$ is the indicator of failure after enrollment by τ under assignment a, for $a = 0, 1$. This assumption implies that conditioning on $Y^{0-\tau}(a) = 0$ and X is the same thing as conditioning on $Y^{0-\tau}(1) = Y^{0-\tau}(0) = 0$ and X, as noted in Shepherd et al. (2006). For arm $A = 1$, it requires that the risk of the target outcome under assignment $A = 1$ is the same in the $\{Y^{0-\tau}(1) = Y^{0-\tau}(0) = 0\}$ always-survivors principal stratum as in the $\{Y^{0-\tau}(1) = 0, Y^{0-\tau}(0) = 1\}$ principal stratum within levels of X

and $S(1)$ (for participants with $Y^{0-\tau} = 0$). A similar interpretation applies for arm $A = 0$. This assumption (e.g., for $A = 1$) can be violated if experiencing the target outcome by τ under control assignment correlates with experiencing the target outcome by τ under treatment assignment, which could occur due to exposure or biological susceptibility factors not fully captured in the baseline covariates X. Further relaxing the assumptions considered above would require additional sensitivity analysis that would further enlarge the estimated uncertainty intervals about treatment efficacy.

C. Mapping of Causal Parameters to Statistical Estimands (Identifiability)

To show identification of $E[Y(0)|Z = 0]$ in equation (4) of the main article, we consider the parameter within levels of baseline covariates X:

$$\begin{aligned}
E[Y(0)|X, Z = 0] &= E[Y(0)|X, Z = 0, A = 0] && \text{(randomization A2)} \\
&= E[E[Y(0)|X, Z = 0, A = 0, S(0)]|X, Z = 0, A = 0] && \text{(iterative expectation)} \\
&= E[E[Y(0)|X, Z = 1, A = 0, S(0)]|X, Z = 0, A = 0] \\
&\qquad - E[\mu^{UC}(X, S(0))|X, Z = 0, A = 0] && \text{(A4, A5)} \\
&= E[g(X, S)|X, Z = 0, A = 0] \\
&\qquad - E[\mu^{UC}(X, S)|X, Z = 0, A = 0]. && \text{(causal consistency A1)}
\end{aligned}$$

By averaging $E[Y(0)|X, Z = 0]$ with respect to the distribution of X conditional on $Z = 0$, we then obtain:

$$\begin{aligned}
E[Y(0)|Z = 0] = E\big\{&E[g(X, S)|X, Z = 0, A = 0] \mid Z = 0\big\} \\
&- E\big\{E[\mu^{UC}(X, S)|X, Z = 0, A = 0] \mid Z = 0\big\}.
\end{aligned}$$

Lastly, from Rose and van der Laan (2011), $g(X, S) := E(Y \mid X, Z = 1, A = 0, S)$ and each $g_a^*(X, S)$, which is defined in the section describing Step 5 of the Causal Roadmap, is identified

by equation (5) of the main article.

For identification of $E[Y(1)|Z=0]$ in equation (6) of the main article, let $H^{01}(s|x)$ be the cdf of $S(1)$ conditional on $X, Z=0, A=1$. Calculations show:

$$
\begin{aligned}
&E[Y(1)|X=x,Z=0] = E[Y(1)|X=x,Z=0,A=1] && \text{(A2)}\\
&= E[E[Y(1)|X=x,Z=0,A=1,S(1)]|X=x,Z=0,A=1] && \text{(iter. exp.)}\\
&= \int E[Y(1)|X=x,Z=0,A=1,S(1)=s]dH^{01}(s|x) && \\
&= \int \big\{E[Y(0)|X=x,Z=0,A=0,S(0)=s] + u^{CT}(x,s)\big\}\, dH^{01}(s|x) && \text{(A2, A3, A6)}\\
&= \int \big\{E[Y(0)|X=x,Z=1,A=0,S(0)=s] + u^{CT}(x,s) - u^{UC}(x,s)\big\}\, dH^{01}(s|x) && \text{(A4, A5)}\\
&= E[E[Y(0)|X=x,Z=1,A=0,S(0)]|X=x,Z=0,A=1] && \\
&\;+ E[\mu^{CT}(x,S)|X=x,Z=0,A=1] - E[\mu^{UC}(x,S)|X=x,Z=0,A=1] && \\
&= E[g(x,Z=1,S)|X=x,Z=0,A=1] && \\
&\;+ E[\mu^{CT}(x,S)|X=x,Z=0,A=1] - E[\mu^{UC}(x,S)|X=x,Z=0,A=1]. && \text{(A1)}
\end{aligned}
$$

The same as done for $E[Y(0)|Z=0]$, by averaging $E[Y(1)|X,Z=0]$ with respect to the distribution of X conditional on $Z=0$, we obtain:

$$
\begin{aligned}
E[Y(1)|Z=0] = {} & E\big\{E[g(X,S)|X,Z=0,A=1] \mid Z=0\big\}\\
& + E\big\{E[\mu^{CT}(X,S) - \mu^{UC}(X,S)|X,Z=0,A=1] \mid Z=0\big\}.
\end{aligned}
$$

Lastly, exactly as for $E[Y(0)|Z=0]$, from Rose and van der Laan (2011), $g(X,S) := E(Y \mid X, Z=1, A=0, S)$ and each $g_a^*(X,S)$ is identified by equation (5) of the main article.

D. Sandwich Variance Estimation for the Plug-in Estimator

In this section, we describe how to calculate the sandwich variance for the plug-in estimators (Stefanski and Boos, 2002). For simplicity, we consider the no-bias function scenario, although our R code allows for the bias functions. Our plug-in estimators are defined as:

$$\widehat{\theta}_{a,\text{plug-in}} = \frac{1}{n_{RCT}} \sum_{i=1}^{n} I(Z_i = 0)\widehat{E}[\widehat{g}(X,S)|X_i, Z_i = 0, A_i = a].$$

To ease notation, we write $g(x,s) := g(X = x, Z = 1, S = s)$ and define $\mu_a(x) := E[g(X,S)|X = x, Z = 0, A = a]$, for $a = 0, 1$. Further, from previously defined notation, we have sampling probabilities $\pi(x,z,a,t,\delta) := P(\epsilon_s = 1|X = x, Z = z, A = a, \tilde{T} = t, \Delta = \delta)$.

Suppose that we have parametric models for nuisance functions g, μ_a, and $\pi(\cdot)$, with parameters β, γ_a, and α, respectively. We therefore write our set of nuisance functions as $g(x,s;\beta)$, $\mu_0(x;\gamma_0)$, $\mu_1(x;\gamma_1)$, and $\pi(x,z,a,t,\delta;\alpha)$. Let $\hat{\beta}$, $\hat{\gamma}_0$, $\hat{\gamma}_1$ and $\hat{\alpha}$ denote estimators for these parameters obtained through estimating equations. First, let h_π denote the estimating function used to obtain $\hat{\alpha}$:

$$0 = \sum_{i=1}^{n} h_\pi(X_i, Z_i, A_i, \epsilon_{Si}, \tilde{T}_i, \Delta_i; \alpha).$$

Next, h_g^C, $h_{\mu_0}^C$, and $h_{\mu_1}^C$ denote the estimating functions used to obtain $\hat{\beta}$, $\hat{\gamma}_0$, and $\hat{\gamma}_1$ assuming complete data. To account for incomplete sampling of S, we use inverse probability sampling (IPS) weighting, which gives us our final estimating equations:

$$0 = \sum_{i=1}^{n} h_g(X_i, S_i, Z_i, A_i, \epsilon_{S_i}, \tilde{T}_i, \Delta_i; \beta, \alpha)$$

$$0 = \sum_{i=1}^{n} h_{\mu_0}(X_i, S_i, Z_i, A_i, \epsilon_{S_i}, \tilde{T}_i, \Delta_i; \alpha, \beta, \gamma_0)$$

$$0 = \sum_{i=1}^{n} h_{\mu_1}(X_i, S_i, Z_i, A_i, \epsilon_{S_i}, \tilde{T}_i, \Delta_i; \alpha, \beta, \gamma_1)$$

where

$$h_g(X_i, S_i, Z_i, A_i, \epsilon_{S_i}, \tilde{T}_i, \Delta_i; \beta, \alpha) := \frac{Z_i \epsilon_{S_i} h_g^C(X_i, S_i, \tilde{T}_i, \Delta_i; \beta)}{\pi(X_i, Z_i, A_i, \tilde{T}_i, \Delta_i; \alpha)}$$

$$h_{\mu_0}(X_i, S_i, Z_i, A_i, \epsilon_{S_i}, \tilde{T}_i, \Delta_i; \alpha, \beta, \gamma_0) := \frac{(1-Z_i)(1-A_i)\epsilon_{S_i} h^C_{\mu_0}(g(X_i, S_i, \tilde{T}_i, \Delta_i; \beta), X_i; \gamma_0)}{\pi(X_i, Z_i, A_i, \tilde{T}_i, \Delta_i; \alpha)}$$
$$h_{\mu_1}(X_i, S_i, Z_i, A_i, \epsilon_{S_i}, \tilde{T}_i, \Delta_i; \alpha, \beta, \gamma_1) := \frac{(1-Z_i)A_i\epsilon_{S_i} h^C_{\mu_1}(g(X_i, S_i, \tilde{T}_i, \Delta_i; \beta), X_i; \gamma_1)}{\pi(X_i, Z_i, A_i, \tilde{T}_i, \Delta_i; \alpha)}.$$

The plug in estimators $\widehat{\theta}_{0,\text{plug-in}}$ and $\widehat{\theta}_{1,\text{plug-in}}$ can be written as the solutions ϕ_0, ϕ_1, respectively, of the following estimating equations:

$$0 = \sum_{i=1}^{n}(1-Z_i)\{\mu_0(X_i;\gamma_0) - \phi_0\}$$
$$0 = \sum_{i=1}^{n}(1-Z_i)\{\mu_1(X_i;\gamma_1) - \phi_1\}.$$

We define the parameter vector $\nu := (\beta, \gamma_0, \gamma_1, \alpha, \phi_0, \phi_1)$. Our final set of stacked estimating functions are

$$h_{\text{stack}}\,(X_i, S_i, Z_i, A_i, \epsilon_{Si}, \tilde{T}_i, \Delta_i; \nu) = \begin{pmatrix} h_\pi(\epsilon_{Si}, X_i, Z_i, A_i, \tilde{T}_i, \Delta_i; \alpha) \\ h_g(X_i, S_i, Z_i, A_i, \epsilon_{S_i}, \tilde{T}_i, \Delta_i; \beta, \alpha) \\ h_{\mu_0}(X_i, S_i, Z_i, A_i, \epsilon_{S_i}, \tilde{T}_i, \Delta_i; \alpha, \beta, \gamma_0) \\ h_{\mu_1}(X_i, S_i, Z_i, A_i, \epsilon_{S_i}, \tilde{T}_i, \Delta_i; \alpha, \beta, \gamma_1) \\ (1-Z_i)\{\mu_0(X_i;\gamma_0) - \phi_0\} \\ (1-Z_i)\{\mu_1(X_i;\gamma_1) - \phi_1\} \end{pmatrix}$$

along with the stacked estimating equation

$$0 = \sum_{i=1}^{n} h_{\text{stack}}\,(X_i, S_i, Z_i, A_i, \epsilon_{Si}, \tilde{T}_i, \Delta_i; \nu).$$

From this estimating equation, we obtain an estimator $\hat{\nu}$. Now, we define the following:

$$h'_{\text{stack}}\,(X_i, S_i, Z_i, A_i, \epsilon_{Si}, \tilde{T}_i, \Delta_i; \nu) = \partial h_{\text{stack}}\,(X_i, S_i, Z_i, A_i, \epsilon_{Si}, \tilde{T}_i, \Delta_i; \nu)/\partial\nu,$$
$$W_n\left(X_i, S_i, Z_i, A_i, \epsilon_{Si}, \tilde{T}_i, \Delta_i; \hat{\nu}\right) = \frac{1}{n}\sum_{i=1}^{n}\left\{-h'_{\text{stack}}\,(X_i, S_i, Z_i, A_i, \epsilon_{Si}, \tilde{T}_i, \Delta_i; \hat{\nu})\right\}$$
$$Q_n\left(X_i, S_i, Z_i, A_i, \epsilon_{Si}, \tilde{T}_i, \Delta_i; \hat{\nu}\right) = \frac{1}{n}\sum_{i=1}^{n} h_{\text{stack}}\,(X_i, S_i, Z_i, A_i, \epsilon_{Si}, \tilde{T}_i, \Delta_i; \hat{\nu}) h_{\text{stack}}\,(X_i, S_i, Z_i, A_i, \epsilon_{Si}, \tilde{T}_i, \Delta_i; \hat{\nu})^T.$$

The sandwich variance estimator for $\hat{\nu}$ is

$$V_n\left(X_i, S_i, Z_i, A_i, \epsilon_{Si}, \tilde{T}_i, \Delta_i; \hat{\nu}\right) = W_n\left(X_i, S_i, Z_i, A_i, \epsilon_{Si}, \tilde{T}_i, \Delta_i; \hat{\nu}\right)^{-1} Q_n\left(X_i, S_i, Z_i, A_i, \epsilon_{Si}, \tilde{T}_i, \Delta_i; \hat{\nu}\right)$$
$$\left\{W_n\left(X_i, S_i, Z_i, A_i, \epsilon_{Si}, \tilde{T}_i, \Delta_i; \hat{\nu}\right)^{-1}\right\}^T.$$

E. Specification of the two bias functions $u^{UC}(X, S)$ and $u^{CT}(X, S)$

How can a plausible range for the Untreated-to-Control transport bias function $u^{UC}(X, S)$, which specifies violation of the Comparability assumption A4, be specified? It will commonly be the case that the observational studies are completed (or almost so) by the the time the phase 3 study collects data. This means the untreated $A = 0$ will not overlap in the two studies, implying it is not possible to control for secular calendar trends in target outcome incidence in Untreated-to-Control-transport. Therefore, an idea for sensitivity analysis is to access external data bases to estimate population-level fluctuations in incidence over calendar time, and to use those results to guide specification of $u^{UC}(X, S)$. For example, if two observational studies are conducted in different calendar periods of follow-up, then a linear regression could be fit for outcome Y on X, S and observational study, and u^{UC} taken to be the point estimate (or confidence limit) of the coefficient for observational study. Dahabreh *and others* (2023) focused on a constant bias function $u^{UC}(X, S) = u^{UC}$, and under this approach u^{UC} could be specified to vary over a range that includes the plausible range of calendar fluctuations from the observational study to the phase 3 study. Conduct of multiple observational studies in different geographic regions/populations would aid specification of u^{UC}.

Another idea considers the similarity of the relationship between X and S in the observational study vs. the phase 3 placebo arm, which can be studied with reasonable precision. Under the premise that $P(X, S|Z = 0, A = 0) \neq P(X, S|Z = 1, A = 0)$ would likely imply $P(Y|X, Z = 0, A = 0, S) \neq P(Y|X, Z = 1, A = 0, S)$, the greater the dissimilarity in the (X, S) relationship the more need to increase the absolute value of the bias function $u^{UC}(X, S)$.

Next, how can a plausible range for the Control-to-Treated transport bias function $u^{CT}(X, S)$, which specifies violation of the Perfect Surrogacy version of A6, be specified? This is challenging given the absence of any previous phase 3 randomized trials to help validate the surrogate, and we have emphasized that in our problem context there are too-few $Y = 1$ outcomes in any

randomized trial to estimate $u^{CT}(X,S)$. However, we know S provides a (very) strong correlate of risk, or otherwise the pathway for provisional approval would have little chance of success, and this strong association provides some likelihood that the marker is at least a partially valid surrogate endpoint. One simple recipe is as follows. Consider X-specific treatment efficacy on the additive difference scale: $\mathrm{TE}^{ATE}(X) = E[Y(1)|X,Z=0] - E[Y(0)|X,Z=0]$. Then the proportion of the treatment effect on the target outcome explained by S (Freedman *and others*, 1992), is $\mathrm{PTE}(X) := 1 - u^{CT}(X,S)/\mathrm{TE}^{ATE}(X)$, where $u^{CT}(X,S)$ is selected to not depend on S. Therefore

$$u^{CT}(X,S) = \mathrm{TE}^{ATE}(X)\,[1 - \mathrm{PTE}(X)]\,. \tag{E.1}$$

Set multiplicative treatment efficacy $\mathrm{TE}(X) = 1 - E[Y(1)|X,Z=0]/E[Y(0)|X,Z=0]$ to the preferred target product profile (TPP) level for the candidate treatment that indicates clear merit for use. Next, set a lower bound for $\mathrm{PTE}(X)$, such as 0.5 (Lin *and others*, 1997), which may be credible given the high correlation of S with Y in the observational study. Lastly, as $\mathrm{TE}^{ATE}(X) = -P(Y(0)=1|X,Z=0)\mathrm{TE}(X)$, the placebo arm disease rate $P(Y(0)=1|X,Z=0)$ is specified, with one choice being the point estimate in the observational study.

For example, suppose $P(Y(0)=1|X,Z=0)$ is set to 0.005 based on the exercise to set a plausible range for $U^{UC}(X,S) = U^{UC}$, and TE is set to 0.7, such that $\mathrm{TE}^{ATE} = -0.0035$. Then, setting $\mathrm{PTE}(X) = 0.5$ yields $u^{CT}(X,S) = -0.00175$, and accordingly $u^{CT}(X,S) = u^{CT}$ could be varied over the range -0.00175 to 0.00175 (allowing imperfection in the surrogate to move the conditional mean difference in A6 an equal distance negative or positive).

Another consideration for specifying $u^{CT}(X,S)$ is that the more unmeasured confounding of the effect of S on Y in the estimation of $g(X,S)$, the more opportunity for Control-to-Treated-transport to fail. Therefore, domain knowledge on the confounders included in X, as well as concerns about missing confounders, influence the range of specified $u^{CT}(X,S)$. To address additional uncertainty sources, additional sensitivity parameters could be specified and varied over

plausible ranges and their variability accounted for in the ignorance intervals and estimated uncertainty intervals.

F. Application of the Provisional Approval Causal Roadmap to the GBS Vaccine Development Case Study: Steps 1–6

F.1 *Step 1: Specify the causal model based on available knowledge of the context and proposed study*

The concentration of IgG antibodies that bind to various GBS surface proteins is highly predictive of the target disease outcome IGbsD in many natural history studies. The OpKA functional assay is believed to measure a causal mechanism of vaccine protection. Thus, some subject matter experts suggest the perfect-surrogate causal model of Figure 2 [Panel (A)] approximately holds. However, one reason the imperfect-surrogate causal model [Panel (B)] is more appropriate is that IgG and OpKA levels for vaccinated and not infected with GBS vs. naturally GBS infected mothers may have different time-patterns in the infant from pre-birth through 90 days of age. A second reason is that the vaccine exposes a mother and baby to a specific GBS protein whereas natural-infection exposes a mother and baby to the entire GBS bacterium, meaning that natural-infection induces additional immune responses, and, if these responses help protect against IGbsD, then the bias function $u^{CT}(X, S)$ would likely be positive.

F.2 *Step 2: Define the causal parameter of interest*

The causal parameter of interest is $\text{VE} = 1 - E[Y(1)|Z = 0]/E[Y(0)|Z = 0]$ with $Y = I(T \leqslant 90)$: vaccine efficacy against IGbsD occurrence through $t_0 = 90$ days of life in the population of live-born infants.

F.3 *Step 5: Estimate the statistical estimand*

We consider the plug-in estimator and the one-step estimator of $E[Y(0)|Z = 0]$, $E[Y(1)|Z = 0]$, and the target parameter TE = VE. For estimating $g(X, S)$ from the sero-epidemiological observational studies, the baseline variables X are taken to be a set of known prognostic risk factors for IGbsD: X_1 the indicator of gestational age less than 37 weeks, and X_2 maternal age in years where younger age is a risk factor. Multivariate S based on cord-blood samples that are considered are based on the four measurements of $\log_{10}$ IgG concentration against each of the four GBS alpha types in the vaccine. In addition to considering the individual S measurements, univariate scores aggregating across the four measurements are considered, including the average, the minimum and maximum, the first and second principal components, non-linear principal components, and the maximum signal diversity weighted average (He and Fong, 2019). As noted in the main article, many different IPS-weighted regression estimators for $g(X, S)$ may be considered. A superlearner estimator has appeal for providing an estimated optimal surrogate (EOS) to use in the estimation formulas, where several EOSs would be developed each under a different set of input variables (X, S), providing an empirical approach to selecting a best-predictive and parsimonious (X, S) to include in the phase 3 surrogate endpoint study data analysis (Price *and others*, 2018). For specification of the phase 3 statistical analysis plan, it would be useful to conduct a simulation study that compares performance of different implementations of the estimators (and variance estimators), not only considering different input variable sets but also different libraries of learners in the superlearner and different implementation details such as loss function and cross-validation scheme.

F.3.1 *Intercurrent events* In the GBS application, $Y^{0-\tau}$ is the indicator of acquiring IGbsD by birth, such that the simplifying assumption $P(Y^{0-\tau} = 1) = 0$ amounts to no in-utero IGbsD that has onset on the date of birth. If such events do occur, then additional investigations would

be warranted to provide evidence for whether restricting the analysis to infants with $Y^{0-\tau} = 0$ would be expected to affect VE in the whole cohort. An alternative framing would redefine $Y^{0-\tau} = 1$ to mean IGbsD onset *before* birth and therefore structurally to make the assumption $P(Y^{0-\tau} = 1) = 0$ hold, in which case S measured from cord blood would need to be considered definable, where some infants may have IGbsD onset at birth that are assigned a failure time of one day. This latter framing may be reasonable based on the fact that infants receive the S immune marker levels passively from the mother.

F.4 *Step 6: Quantify the uncertainty in the estimate of the statistical estimand*

It is of interest to compare the two variance estimation approaches for the plug-in estimator (sandwich, bootstrap) for the specific context of the planned/available GBS sero-epidemiological studies and potential phase 3 study designs. These two variance estimators were evaluated in the simulation study. It is also of interest to study strategies for specifying the bias functions.

G. Simulation Study Details and Additional Simulation Studies for the GBS Vaccine Development Case Study

G.1 *Design of simulation study conditions*

The simulation study was designed to approximately match published characteristics of GBS and its risk factors.

1. Probability of IGbsD by 90 days of age ≈ 0.005 (Vekemans *and others*, 2019).

2. Geometric mean (95% CI) of cord-blood IgG concentration 0.01 (0.01-0.02) in IGbsD cases and 0.04 (0.03 - 0.06) in controls, which was observed for infant RibN IgG in Dangor *and others* (2023).

3. Covariates X_1, X_2, X_3, where X_1 represents pre-term birth (less than 37 weeks gestational

age), X_2 represents maternal age (younger age a risk factor), and X_3 represents a continuous covariate unrelated to the outcome.

$$X_1|Z, A \sim \text{Bernoulli}(0.05), X_2|Z, A \sim \text{Uniform}(18, 40), X_3|Z, A \sim \text{Normal}(0, 1)$$

- Pre-term birth is associated with roughly 2.56× odds of early onset IGbsD (Puopolo *and others*, 2011).
- Maternal age < 25 years is associated with roughly 1.94× odds of IGbsD (Parente *and others*, 2017).

Based on these constraints, we set the following to be our true $A = 0$ data generating conditional regression function:

$$P(Y = 1|S, X_1, X_2, X_3, A = 0, Z) = \beta_0 + \beta_1 S + \beta_2 X_1 + \beta_3 X_2 + \beta_4 X_3$$

where $\beta_0 = -17.1$, $\beta_1 = -8.2$, $\beta_2 = 0.69$, $\beta_3 = -0.03$, $\beta_4 = 0$, and we set our distribution of $S \sim \text{Normal}(-1.45, 0.0225)$, where S represents the log IgG biomarker, $X_1 \sim \text{Bernoulli}(0.05)$, $X_2 \sim \text{Uniform}(18, 40)$, and $X_3 \sim \text{Normal}(0, 1)$.

With this data-generating function, we have that in our observational study (using empirical measures from a simulated n =10,000,000 size data set):

- Incidence of IGbsD by 90 days ≈ 0.005
- Geometric mean cord-blood IgG biomarker in IGbsD cases is 0.01 and in controls is 0.04
- Pre-term birth associated with 2× odds of disease
- Each one-year increase in maternal age associated with 3% lower odds of disease

G.1.1 *Results from the main simulation study*

Table 1. Results for Simulation Study 1 on empirical bias, median standard error, standard deviation of estimates, and 95% confidence interval coverage for $E[Y(0)|Z=0], E[Y(1)|Z=0]$. SE (bs) = bootstrap standard error for plug-in estimator, SE (sw) = sandwich standard error for plug-in estimator, SE (os) = standard error for one-step estimator, SD = standard deviation of estimates, Cov = 95% confidence interval coverage using sandwich standard error, Sampled = number with S data per treatment arm in the phase 3 study. For the VE entries the standard errors shown are for $\log(1-\widehat{\text{VE}})$.

	Plug-In Estimator					One-Step Estimator			
	Bias	SE (bs)	SE (sw)	SD	Cov	Bias	SE (os)	SD	Cov
Sampled = 100									
$E[Y(0)\|Z=0]$	-0.000060	0.000946	0.000917	0.00109	0.9	-0.000080	0.000839	0.00112	0.9
$E[Y(1)\|Z=0]$	-0.000060	0.000954	0.000928	0.00114	0.89	-0.00012	0.000827	0.00115	0.87
VE	-0.00525	0.253	0.232	0.277	0.93	-0.00124	0.227	0.293	0.91
Sampled = 250									
$E[Y(0)\|Z=0]$	0.000030	0.00071	0.000735	0.000805	0.95	-0.0000450	0.000671	0.000876	0.9
$E[Y(1)\|Z=0]$	0	0.00073	0.000734	0.000842	0.95	-0.000010	0.000662	0.000894	0.93
VE	-0.00509	0.169	0.16	0.176	0.94	-0.0147	0.166	0.197	0.93
Sampled = 500									
$E[Y(0)\|Z=0]$	0.000020	0.000581	0.000631	0.000646	0.95	-0.000080	0.000555	0.000705	0.9
$E[Y(1)\|Z=0]$	-0.000050	0.000586	0.000631	0.000642	0.96	-0.0000850	0.000564	0.00071	0.92
VE	-0.00437	0.122	0.116	0.124	0.94	-0.00582	0.127	0.147	0.93
True VE = 0.5									
Sampled = 100									
$E[Y(0)\|Z=0]$	-0.000070	0.000956	0.000918	0.00115	0.9	-0.00012	0.000851	0.00117	0.91
$E[Y(1)\|Z=0]$	-0.000115	0.000642	0.000606	0.00103	0.87	-0.00016	0.000688	0.00121	0.89
VE	0.015	0.321	0.293	0.364	0.93	0.0265	0.344	0.438	0.93
Sampled = 250									
$E[Y(0)\|Z=0]$	-0.0000150	0.000713	0.000725	0.000824	0.95	-0.000060	0.000658	0.000921	0.91
$E[Y(1)\|Z=0]$	0	0.000509	0.000484	0.000582	0.91	-0.00014	0.000532	0.000732	0.88
VE	0.003	0.217	0.204	0.228	0.93	0.0225	0.252	0.294	0.93
Sampled = 500									
$E[Y(0)\|Z=0]$	0.000010	0.000591	0.00064	0.000639	0.96	-0.000060	0.000568	0.000723	0.9
$E[Y(1)\|Z=0]$	-0.000010	0.000392	0.000398	0.000439	0.95	-0.00010	0.000455	0.00060	0.91
VE	0.004	0.166	0.157	0.164	0.96	0.0125	0.202	0.22	0.95
True VE = 0.9									
Sampled = 100									
$E[Y(0)\|Z=0]$	0	0.000939	0.000917	0.0012	0.91	-0.000080	0.000861	0.00127	0.89
$E[Y(1)\|Z=0]$	-0.0000460	0.00014	0.000138	0.000213	0.85	0.0000710	0.000254	0.000413	0.88
VE	0.007	0.386	0.356	0.425	0.92	-0.016	0.476	0.656	0.85
Sampled = 250									
$E[Y(0)\|Z=0]$	0.000010	0.000714	0.00073	0.000826	0.93	-0.00010	0.000685	0.000927	0.9
$E[Y(1)\|Z=0]$	-0.000020	0.000117	0.000119	0.000171	0.89	0.000116	0.000235	0.000383	0.9
VE	0.003	0.293	0.274	0.331	0.92	-0.023	0.398	0.597	0.82
Sampled = 500									
$E[Y(0)\|Z=0]$	0.000020	0.000594	0.000636	0.000641	0.97	-0.000060	0.00059	0.000731	0.92
$E[Y(1)\|Z=0]$	-0.000030	0.0000989	0.000102	0.000116	0.91	0.000112	0.000219	0.000344	0.89
VE	0.007	0.248	0.233	0.253	0.95	-0.023	0.357	0.595	0.82

Table 2. Results for Simulation Study 2 on empirical bias, median standard error, standard deviation of estimates, 95% confidence interval coverage of $E[Y(0)|Z=0], E[Y(1)|Z=0]$, and VE, along with success probabilities. SE (bs) = bootstrap standard error, SE (sw) = sandwich standard error, SD = standard deviation of estimates, Cov = 95% confidence interval coverage using sandwich standard errors, PTE = proportion of treatment explained used to generate bias functions, SP = success probability (criterion defined as 95% EUI for VE $\geqslant 0.3$). For the VE entries the standard errors shown are for $\log(1-\widehat{\mathrm{VE}})$. The S data are sampled from 250 participants in each treatment arm of the phase 3 study.

	Plug-In Estimator						One-Step Estimator				
	Bias	SE (bs)	SE (sw)	SD	Cov	SP	Bias	SE (os)	SD	Cov	SP
PTE = 1											
$E[Y(0)\|Z=0]$	0.000030	0.00071	0.000735	0.000805	0.95		-0.0000450	0.000671	0.000876	0.9	
$E[Y(1)\|Z=0]$	0	0.00073	0.000734	0.000842	0.95		-0.000010	0.000662	0.000894	0.93	
VE	-0.00509	0.169	0.16	0.176	0.94	0	-0.0147	0.166	0.197	0.93	0
PTE = 0.83											
$E[Y(0)\|Z=0]$	-0.000010	0.00071	0.000732	0.000797	0.94		-0.00010	0.000659	0.000875	0.91	
$E[Y(1)\|Z=0]$	0.000645	0.000728	0.000744	0.000811	0.94		0.00057	0.000664	0.000868	0.88	
VE	-0.134	0.158	0.148	0.158	0.86	0	-0.133	0.154	0.177	0.84	0
PTE = 0.67											
$E[Y(0)\|Z=0]$	0.000050	0.000729	0.00074	0.00080	0.94		-0.000030	0.000656	0.000828	0.91	
$E[Y(1)\|Z=0]$	0.00118	0.000708	0.000728	0.000793	0.73		0.00115	0.000656	0.000852	0.61	
VE	-0.222	0.155	0.145	0.149	0.68	0	-0.229	0.15	0.167	0.66	0
True VE = 0.5											
PTE = 1											
$E[Y(0)\|Z=0]$	-0.0000150	0.000713	0.000725	0.000824	0.95		-0.000060	0.000658	0.000921	0.91	
$E[Y(1)\|Z=0]$	0	0.000509	0.000484	0.000582	0.91		-0.00014	0.000532	0.000732	0.88	
VE	0.003	0.217	0.204	0.228	0.93	0.4	0.0225	0.252	0.294	0.93	0.35
PTE = 0.83											
$E[Y(0)\|Z=0]$	0	0.000706	0.000721	0.000821	0.93		-0.000090	0.000648	0.000881	0.91	
$E[Y(1)\|Z=0]$	0.00055	0.000481	0.000467	0.000609	0.9		0.000475	0.000532	0.000744	0.88	
VE	-0.109	0.191	0.179	0.216	0.77	0.14	-0.0935	0.212	0.265	0.82	0.14
PTE = 0.67											
$E[Y(0)\|Z=0]$	0.000040	0.000736	0.000753	0.000816	0.96		-0.0000150	0.000662	0.000939	0.92	
$E[Y(1)\|Z=0]$	0.00118	0.00050	0.000488	0.000649	0.34		0.00105	0.000546	0.000843	0.54	
VE	-0.228	0.181	0.17	0.196	0.43	0.02	-0.213	0.198	0.232	0.54	0.03
True VE = 0.9											
PTE = 1											
$E[Y(0)\|Z=0]$	0.000010	0.000714	0.00073	0.000826	0.93		-0.00010	0.000685	0.000927	0.9	
$E[Y(1)\|Z=0]$	-0.000020	0.000117	0.000119	0.000171	0.89		0.000116	0.000235	0.000383	0.9	
VE	0.003	0.293	0.274	0.331	0.92	1	-0.023	0.398	0.597	0.82	0.92
PTE = 0.83											
$E[Y(0)\|Z=0]$	0.000010	0.000722	0.000737	0.000807	0.94		-0.000070	0.000693	0.000933	0.92	
$E[Y(1)\|Z=0]$	0.00058	0.000118	0.00012	0.000155	0.01		0.00071	0.000236	0.000435	0.19	
VE	-0.115	0.179	0.173	0.203	0.05	0.99	-0.14	0.235	0.398	0.18	0.88
PTE = 0.67											
$E[Y(0)\|Z=0]$	0.0000150	0.000725	0.000736	0.000852	0.94		-0.000060	0.000704	0.000924	0.91	
$E[Y(1)\|Z=0]$	0.00116	0.000117	0.000119	0.000151	0		0.00128	0.000238	0.000541	0.05	
VE	-0.225	0.16	0.158	0.177	0	0.99	-0.251	0.196	0.289	0.02	0.82

Table 3. Results for Simulation Study 3 on empirical bias, median standard error, standard deviation of estimates, 95% confidence interval coverage of $E[Y(0)|Z=0], E[Y(1)|Z=0]$, and VE, along with success probabilities. SE (bs) = bootstrap standard error, SE (sw) = sandwich standard error, SD = standard deviation of estimates, Cov = 95% confidence interval coverage using sandwich standard errors, PTE = proportion of treatment explained used to generate bias functions, SP = success probability (criterion defined as 95% EUI for VE $\geqslant 0.3$). For the VE entries the standard errors shown are for $\log(1-\widehat{\text{VE}})$. The S data are sampled from 250 participants in each treatment arm of the phase 3 study.

	Plug-In Estimator						One-Step Estimator				
	Bias	SE (bs)	SE (sw)	SD	Cov	SP	Bias	SE (os)	SD	Cov	SP
True VE = 0											
$E[Y(0)\|Z=0]$	0.000010	0.000239	0.000428	0.000377	0.97		-0.0000050	0.000391	0.000385	0.96	
$E[Y(1)\|Z=0]$	0	0.00024	0.000428	0.000372	0.98		-0.000010	0.00039	0.000388	0.96	
VE	-0.00111	0.0418	0.0398	0.0406	0.97	0	-0.00164	0.0393	0.0565	0.96	0
True VE = 0.5											
$E[Y(0)\|Z=0]$	-0.000020	0.000238	0.000427	0.000393	0.97		-0.0000550	0.000379	0.000399	0.94	
$E[Y(1)\|Z=0]$	0.00075	0.00027	0.000354	0.000329	0.42		-0.000050	0.000498	0.000592	0.94	
VE	-0.148	0.087	0.0854	0.0857	0.2	0.11	0.0045	0.184	0.233	0.93	0.46
True VE = 0.9											
$E[Y(0)\|Z=0]$	0.000040	0.00024	0.00043	0.000416	0.96		-0.000060	0.000377	0.000432	0.91	
$E[Y(1)\|Z=0]$	0.00198	0.000315	0.000356	0.000329	0		0.0000320	0.000385	0.000449	0.96	
VE	-0.388	0.133	0.13	0.129	0	0.84	-0.006	0.675	0.828	0.92	0.68

G.2 *Results from the larger sample simulation study*

In this section, we report results for a simulation study with a higher case rate. We set the following to be our true $A=0$ data generating conditional regression function:

$$P(Y=1|X_1, X_2, X_3, A=0, Z, S) = \beta_0 + \beta_1 S + \beta_2 X_1 + \beta_3 X_2 + \beta_4 X_3$$

where $\beta_0 = -14$, $\beta_1 = -7$, $\beta_2 = 0.69$, $\beta_3 = -0.03$, $\beta_4 = 0$.

We preserve the same distribution of S as in Simulation Study 1, with $S \sim \text{Normal}(-1.45, 0.225)$, resulting in a baseline case rate of 0.016 (about 3 times higher than 0.005).

To generate VE $= \{0, 0.5, 0.9\}$, we manipulate the distribution of the biomarker in the vaccine arm $S|A=1, Z=0$:

- To create VE $= 0$, set $S|A=1, Z=0 \sim \text{Normal}(-1.45, 0.0225)$
- To create VE $= 0.5$, set $S|A=1, Z=0 \sim \text{Normal}(-1.29, 0.04)$
- To create VE $= 0.9$, set $S|A=1, Z=0 \sim \text{Normal}(-1.04, 0.0441)$.

Table 4. Results for the Supplemental Simulation Study with approximately 3 times higher IGbsD outcome rate (expect 585 invasive GBS disease cases by 90 days of age) on empirical bias, median standard error, standard deviation of estimates, and 95% confidence interval coverage of $E[Y(0)|Z=0]$, $E[Y(1)|Z=0]$, and VE. SE (bs) = bootstrap standard error, SE (sw) = sandwich standard error, SD = standard deviation of estimates, Cov = 95% confidence interval coverage using sandwich standard error. For the VE entries the standard errors shown are for $\log(1-\widehat{\text{VE}})$. The S data are sampled from 250 participants in each treatment arm of the phase 3 study.

	Plug-In Estimator					One-Step Estimator			
	Bias	SE (bs)	SE (sw)	SD	Cov	Bias	SE (os)	SD	Cov
True VE = 0									
$E[Y(0)\|Z=0]$	0	0.00136	0.00146	0.00147	0.95	0	0.00136	0.00146	0.94
$E[Y(1)\|Z=0]$	-0.000050	0.00136	0.00146	0.00147	0.96	-0.00010	0.00136	0.00145	0.94
VE	0.00623	0.117	0.115	0.121	0.95	0.00346	0.11	0.12	0.94
True VE = 0.5									
$E[Y(0)\|Z=0]$	-0.00010	0.00136	0.00146	0.0015	0.96	-0.00010	0.00136	0.0015	0.94
$E[Y(1)\|Z=0]$	-0.000060	0.00101	0.00103	0.0011	0.93	-0.00015	0.00101	0.00115	0.92
VE	0	0.152	0.148	0.154	0.94	0.006	0.15	0.161	0.95
True VE = 0.9									
$E[Y(0)\|Z=0]$	-0.00010	0.00136	0.00146	0.00148	0.94	-0.00020	0.00135	0.00149	0.93
$E[Y(1)\|Z=0]$	-0.000040	0.000255	0.00026	0.000292	0.92	0.00011	0.000361	0.000487	0.93
VE	0.003	0.193	0.189	0.202	0.95	-0.0085	0.233	0.283	0.89

Finally, matching the simulations in the main article, we sample 250 participants from each of the vaccine and placebo arms for measurement of S in the phase 3 study ($Z=0$).

Results are shown in Supplementary Table 4. In this higher case rate scenario, we observe minimal bias in estimating VE, approximately nominal confidence interval coverage, and close agreement between bootstrap, sandwich, and empirical standard errors.

G.3 *Vignette Application to a Single Data Set*

We provide a simulated observational study dataset and phase 3 study dataset as well as R code for running the analysis on this dataset in the GitHub repository here.

The simulated observational study dataset `sim_obs_data.csv` and phase 3 study dataset `sim_p3_data.csv` include the following variables:

- Outcome: `Y` (taking values 0 or 1)
- Covariates: `X1, X2, X3`

- Surrogate: univariate surrogate `S`
- Treatment: `A` (taking values 0 or 1)

The function for analyzing a single dataset is `estimate_ve_surrogate()`. The function estimates $E[Y(0)|Z=0]$, $E[Y(1)|Z=0]$, and $\text{VE} = 1 - E[Y(1)|Z=0]/E[Y(0)|Z=0]$, as well as variance estimates and confidence intervals, over all combination of u^{CT} and u^{UC} constant bias functions specified by the user. It also prints results for the tipping-point analysis summarized in the main article. To perform this tipping-point analysis, we first set $u^{UC}=0$, and calculate the highest magnitude positive value of u^{CT} under which the analysis just barely meets the success criterion (at the boundary of the success criterion). Then, setting $u^{CT}=0$, we find the highest magnitude negative value of u^{UC} for which the success criterion is just barely attained. The success criterion can be defined by either a minimum required lower confidence bound for VE, a minimum required point estimate for VE, or requiring both. The tipping-point analysis is not conducted if the success criterion is not attained when both bias functions equal 0, i.e. $u^{CT}=0$ and $u^{UC}=0$.

The `estimate_ve_surrogate()` function takes in the following inputs:

- `df_obs`: `data.frame` for observational study dataset
- `df_p3`: `data.frame` for phase 3 study dataset
- `treatment`: String for binary treatment (0/1) variable name
- `outcome`: String for binary outcome (0/1) variable name with no missing data
- `covariates`: Vector of covariate variable names
- `surrogate`: String for surrogate variable name (assuming a univariate surrogate)
- `estimate_weights`: Boolean indicator for whether sampling weights should be estimated or if they are included as a column in the data
- `weight`: If `estimate_weights = F`, then indicate column name for weights. The weights should be inverse probability sampling weights with values greater than or equal to 1.

- `estimator`: Either "plugin" or "onestep". The plug-in estimator uses bootstrapping to obtain variance estimates. The one-step estimator estimates each variance using the empirical variance of the efficient influence function computed across all observations.
- `learner`: Either "glm" or "superlearner" is used to specify how nuisance functions are estimated. The superlearner defaults to use the `SL.mean`, `SL.glm`, and `SL.gam` in the ensemble learner. Note that the plugin estimator only allows for "glm" estimation of nuisance functions.
- `ct_bias_values`: Vector of u^{CT} constant bias functions. $u^{CT} = 0$ is included automatically.
- `uc_bias_values`: Vector of u^{UC} constant bias functions. $u^{UC} = 0$ is included automatically.
- `alpha`: A value between 0 and 1 specifying the significance level for a $(1 - \alpha)\%$ two-sided confidence intervals. Default value is 0.05.
- `success_criterion`: A vector of two values. The two values specify the minimum lower confidence bound of a (1-`alpha`)% two-sided confidence interval and minimum point estimate of VE, respectively, to define the success criterion. Specify `NA` to exclude one of the two criteria. For example, if we set `success_criterion = c(0.3, NA)`, then we are specifying the success criterion to be that the lower confidence bound for VE is greater than 0.3.
- `bootstrap_reps`: Number of bootstrap replications used to estimate variances (for the plug-in estimator only).

The function outputs a `data.frame` with the results for each combination of u^{UC} and u^{CT} bias values specified. This `data.frame` includes the following columns:

- `ct_bias`: specified constant u^{CT} bias function used in the analysis
- `uc_bias`: specified constant u^{UC} bias function used in the analysis
- `Y_1, Y_1_se, Y_1_lower, Y_1_upper`: Point estimate, estimated standard error of $\widehat{E}[Y(1)|Z = 0]$, and (1-`alpha`)% two-sided confidence interval for $E[Y(1)|Z = 0]$
- `Y_0, Y_0_se, Y_0_lower, Y_0_upper`: Point estimate, estimated standard error of $\widehat{E}[Y(0)|Z =$

0], and (1-`alpha`)% two-sided confidence interval for $E[Y(0)|Z=0]$

- `log_1_minus_VE, log_1_minus_VE_se`: Point estimate and estimated standard error of $\log(1-\widehat{\text{VE}})$
- `VE, VE_lower, VE_upper`: Point estimate and (1-`alpha`)% two-sided confidence interval for VE

Input to conduct analysis on the simulated datasets:

```
estimate_ve_surrogate(df_obs,
                      df_p3,
                      treatment = "A",
                      outcome = "Y",
                      covariates = c("X1", "X2", "X3"),
                      surrogate = "S",
                      estimate_weights = T,
                      weight = NULL,
                      estimator = "onestep",
                      learner = "glm",
                      ct_bias_values = c(0,0.001),
                      uc_bias_values = c(0,-0.001),
                      alpha = 0.05,
                      success_criterion = c(0.3, NA))
```

Output (some columns not excluded for presentation purposes):

```
  ct_bias uc_bias         Y_1         Y_0        VE log_1_minus_VE      Y_1_se       Y_0_se
1   0.000   0.000 0.002615192 0.005288869 0.5055290     -0.7042667 0.000228638 0.0004161404
2   0.001   0.000 0.003615192 0.005288869 0.3164526     -0.3804593 0.000228638 0.0004161404
```

```
3   0.000  -0.001 0.003615192 0.006288869 0.4251442     -0.5536361 0.000228638 0.0004161404
4   0.001  -0.001 0.004615192 0.006288869 0.2661331     -0.3094276 0.000228638 0.0004161404
```

Additionally, the function prints the results of the tipping point analysis:

```
-----------------------------
 Tipping Point Analysis
Lower VE CI = 0.3 Threshold
-----------------------------
 CT Bias:  0.0005386195
 UC Bias:  -0.001770816
```

In this simulated dataset, the tipping point analysis indicates that if $u^{UC} = 0$, then $u^{CT} > 0.000539$ will cause the lower 2-sided 95% lower confidence bound for VE to be less than 0.3. Similarly, if the true $u^{CT} = 0$, then $u^{UC} < -0.00177$ will cause the lower 2-sided 95% lower confidence bound for VE to be less than 0.3.

References

Athey, S, Chetty, R, Imbens, GW and Kang, H. (2024). The surrogate index: Combining short-term proxies to estimate long-term treatment effects more rapidly and precisely. *National Bureau of Economic Research Working Papers* **Working Paper 26463 updated April 2024**.

Dahabreh, Issa J, Robins, James M, Haneuse, Sebastien J-PA, Saeed, Iman, Robertson, Sarah E, Stuart, Elizabeth A and Hernán, Miguel A. (2023). Sensitivity analysis using bias functions for studies extending inferences from a randomized trial to a target population. *Statistics in Medicine* **42**(13), 2029–2043.

Dangor, Ziyaad, Kwatra, Gaurav, Pawlowski, Andrzej, Fisher, Per B, Izu, Alane,

Lala, Sanjay G, Johansson-Lindbom, Bengt and Madhi, Shabir A. (2023). Association of infant Rib and Alp1 surface protein N-terminal domain immunoglobulin G and invasive Group B Streptococcal disease in young infants. *Vaccine* **41**(10), 1679–1683.

Freedman, LS, Graubard, BI and Schatzkin, A. (1992). Statistical validation of intermediate endpoints for chronic diseases. *Statistics in Medicine* **11**, 167–178.

Gilbert, Peter B, Blette, Bryan S, Shepherd, Bryan E and Hudgens, Michael G. (2020). Post-randomization biomarker effect modification analysis in an HIV vaccine clinical trial. *Journal of Causal Inference* **8**(1), 54–69.

He, Zonglin and Fong, Youyi. (2019). Maximum diversity weighting for biomarkers with application in HIV-1 vaccine studies. *Statistics in Medicine* **38**(20), 3936–3946.

Lin, DY, Fleming, TR and De Gruttola, V. (1997). Estimating the proportion of treatment effect explained by a surrogate marker. *Statistics in Medicine* **16**, 1515–1527.

Parente, Victoria, Clark, Reese H, Ku, Lawrence, Fennell, Courtney, Johnson, Makaela, Morris, Emma, Romaine, Andrew, Utin, Uty, Benjamin, Daniel K, Messina, Julia A *and others*. (2017). Risk factors for group b streptococcal disease in neonates of mothers with negative antenatal testing. *Journal of Perinatology* **37**(2), 157–161.

Price, Brenda L, Gilbert, Peter B and van der Laan, Mark J. (2018). Estimation of the optimal surrogate based on a randomized trial. *Biometrics* **74**(4), 1271–1281.

Puopolo, Karen M, Draper, David, Wi, Soora, Newman, Thomas B, Zupancic, John, Lieberman, Ellice, Smith, Myesha and Escobar, Gabriel J. (2011). Estimating the probability of neonatal early-onset infection on the basis of maternal risk factors. *Pediatrics* **128**(5), e1155–e1163.

Rose, Sherri and van der Laan, Mark J. (2011). A targeted maximum likelihood estimator for two-stage designs. *The International Journal of Biostatistics* **7**(1), 1–21.

Shepherd, B, Gilbert, Peter B, Jemiai, Y and Rotnitzky, A. (2006). Sensitivity analyses comparing outcomes only existing in a subset selected post-randomization, conditional on covariates, with application to HIV vaccine trials. *Biometrics* **62**, 332–342.

Stefanski, Leonard A and Boos, Dennis D. (2002). The calculus of M-estimation. *The American Statistician* **56**(1), 29–38.

Vekemans, Johan, Moorthy, Vasee, Friede, Martin, Alderson, Mark R, Sobanjo-Ter Meulen, Ajoke, Baker, Carol J, Heath, Paul T, Madhi, Shabir A, Mehring-Le Doare, Kirsty, Saha, Samir K *and others*. (2019). Maternal immunization against Group B Streptococcus: World Health Organization research and development technological roadmap and preferred product characteristics. *Vaccine* **37**(50), 7391–7393.
